# Identification of genetic loci for growth and stem form traits in hybrid *Liriodendron* via a genome-wide association study

**DOI:** 10.48130/forres-0025-0001

**Published:** 2025-01-22

**Authors:** Fengchao Zhang, Xiao Liu, Hui Xia, Hainan Wu, Yaxian Zong, Huogen Li

**Affiliations:** State Key Laboratory of Tree Genetics and Breeding, Co-Innovation Center for Sustainable Forestry in Southern China, Nanjing Forestry University, Nanjing 210037, China

**Keywords:** Growth, Stem, Genome-wide association study, Hybrid *Liriodendron*, Candidate genes

## Abstract

A key objective of forest tree breeding programs is to enhance traits related to growth and stem form, to cultivate plantations that exhibit rapid growth, straight trunks with minimal taper, and superior wood quality to meet the demands of modern timber production. Notably, *Liriodendron* species exhibit notable heterosis in interspecies hybrids, with hybrid *Liriodendron* displaying rapid growth rates, straight trunks, and wide adaptability. However, the genetic architecture underlying growth and stem form traits remains unclear, hindering the progress of genetic improvement efforts. Genome-wide association study (GWAS) emerges as an effective approach for identifying target genes and clarifying genetic architectures. In this study, a comprehensive analysis was conducted using an artificial population of 233 hybrid progeny derived from 25 hybrid combinations and resequenced to obtain genome-wide single nucleotide polymorphism (SNP) and insertion and deletion (InDel) variants. After filtering, a total of 192,972 SNP loci and 60,666 InDel loci were obtained, which were subsequently analyzed for associations using the R package GAPIT. We identified 97 significant SNP loci and 58 significant InDel loci (−Log_10_(P) ≥ 4.50), respectively, culminating in the identification of 161 candidate genes. The functions of these candidate genes were annotated, revealing potential associations between *Lchi_2g03172* and *Lchi_10g19986* genes with the growth of hybrid *Liriodendron*, and highlighting the potential influence of the *Lchi_16g30522* gene on the growth and branching of hybrid *Liriodendron*. Overall, this study serves as a foundational step towards unraveling the genetic architecture underpinning growth and stem form in *Liriodendron* plants.

## Introduction

Growth and stem form traits are critical economic attributes with significant implications for timber production in forest trees. The crown, which serves as the primary apparatus for photosynthesis in trees, plays a vital role in influencing tree growth and wood production. Traits such as clear bole height and crown length ratio are essential for assessing crown development in forest trees and provide key indicators of their growth status. In practice, cultivating trees with straight and sturdy trunks is a major breeding objective. Traits such as trunk straightness, degree of forking, branching angle, and branch number are commonly used to evaluate trunk shape. This evaluation process seeks to identify trees with straight trunks, minimal knots, and high timber production potential. These traits are essential for selecting trees that align with desired production outcomes.

*Liriodendron* plants, also known as tulip trees, belong to the genus *Liriodendron* within the Magnoliaceae family and comprise only two natural species: *L. chinense* and *L. tulipifera*. Known for its graceful tree form, distinctive leaf shape, vibrant flowers, and easily processed wood, *Liriodendron* plants serve as both ornamental trees and a valuable industrial timber species^[1]^. The interspecies hybrids between two *Liriodendron* species exhibit distinct advantages in traits such as growth, ornamental value, and adaptability. For instance, Xia et al. conducted research on the progeny of 57 hybrid combinations of six *Liriodendron* parents, studying dynamic changes in genetic parameters of growth traits over time. This study provided insights into parental pair/combinations selection and heterosis prediction, thus expediting genetic improvement processes for *Liriodendron*^[2]^. Forest trees typically have long lifespans and are influenced by various environmental factors during growth^[3]^, leading to genotype-by-environment interactions that affect individual tree phenotypes^[4]^. In a previous study on hybrid *Liriodendron* across three sites, genotype × environment interactions were examined, identifying four superior family lines in terms of growth and adaptability^[5]^. Moreover, previous studies mainly focused on growth traits and rarely on stem-form traits that impact directly on timber quality.

The advent of Next-Generation Sequencing (NGS) technology has ushered in a new era for molecular breeding in forest trees. Genome-wide association study (GWAS) using single nucleotide polymorphism (SNP) molecular markers has emerged as a widely recognized method with vast potential for elucidating the genetic basis of complex traits in forest trees^[6]^. Additionally, linkage analysis or linkage disequilibrium (LD) mapping leverages the diverse phenotypic and genomic variations within natural populations to pinpoint target genes governing complex quantitative traits in forest trees^[7]^. GWAS has gained widespread acceptance for unraveling genetic architecture in various crops such as soybean^[8]^, and in forest trees, including poplar (*Populus* L.)^[9−11]^, eucalyptus (*Eucalyptus* spp.)^[12−14]^, loblolly pine (*Pinus taeda* L.)^[15,16]^, and spruce (*Picea asperata* Mast.)^[17]^. Chen et al. achieved the first complete sequencing and assembly of the Chinese tulip tree genome^[18]^. Subsequently, Xia et al. conducted resequencing of 233 hybrid progenies from 25 cross combinations of Chinese tulip trees, measuring tree height and diameter at breast height over 11 consecutive years, then performing a GWAS using SNPs, revealing 62 SNPs related to tree height and 52 related to diameter at breast height^[19]^.

SNPs are widely used in association analysis due to their high density and abundance. However, SNPs do not represent all types of variants in the genome, and InDels, which are the second most common type of variant after SNPs in terms of distribution and density, alter plant traits by disrupting gene regulatory or coding regions^[20]^. When an InDel occurs in coding regions or splice sites, it may lead to alterations in protein structure and function, which can affect the traits of the organism^[21]^. Some researchers utilized InDels to reveal the genetic mechanisms underlying important plant traits. For example, Hu et al. conducted a GWAS on oilseed rape using InDels and identified 628 locus-related candidate genes for 56 agronomically important traits^[22]^. Therefore, the use of InDel loci for GWAS and candidate gene screening can be cross-validated with SNP loci and serve as a complementary approach^[22,23]^.

In this study, we focused on the growth and stem form traits of hybrid *Liriodendron* and conducted GWAS using various association models on an artificial population derived from multiple hybrid combinations. We aimed to identify SNP and InDel loci and to further determine candidate genes influencing growth and stem form traits in *Liriodendron* plants. Our findings may serve as a foundational step towards underlying the genetic architecture underpinning growth and stem form traits in *Liriodendron* plants.

## Materials and methods

### Plant material

Fourteen adult trees from a provenance testing plantation were used as mating parents, and their offspring were acquired and tested in a progeny trial plantation. Both plantations are located in Xiashu Town, Jurong City, Jiangsu Province, China (latitude 32°12' N, longitude 119°23' E), with an average altitude of 103 m, an average annual temperature of 15.2 °C, and an average annual precipitation of 1,055.6 mm. The soil type is yellow-brown loam, which is suitable for the growth of hybrid *Liriodendron*. The progeny testing plantation was established in 2008 using a randomized block design with four replications and a plant spacing of 4 m × 4 m. From this progeny population, 233 progenies from 25 cross combinations were selected as an experimental population for whole-genome resequencing and phenotyping. The data obtained were used for subsequent GWAS. The hybrid combinations (MSL × WYS and MSL × S) with significant differences in heterosis for growth were selected for RNA-seq by analyzing the variance in growth of the progeny determination stands and estimating the genetic parameters. Detailed information on the experimental population has been described in previous studies^[2]^.

### DNA extraction, resequencing, and genotyping

In July 2021, healthy and fresh leaves were collected from 233 individual forest trees, placed in liquid nitrogen, and stored in a cryogenic refrigerator at −80 °C after being transported to the laboratory. DNA was extracted using the Plant Genomic DNA Extraction Kit (DP320-03) from Tiangen Biologicals (Beijing, China). The DNA was examined using 1% gel electrophoresis and analyzed with a UV spectrophotometer (NanoDrop 2000; Thermo Fisher Scientific, Waltham, MA, USA), and the genomic DNA was used to construct DNA fragment libraries using the TruSeq Nano DNA LT Sample Preparation Kit (Illumina, Inc. San Diego, CA, USA) and sequenced with double ends (PE) at 150 bp per read length on an Illumina HiSeq 4000 platform (OE Biotech. Co., Ltd, Shanghai, China).

Subsequently, the FASTP software^[24]^ was used to filter the raw data for high-quality clean data, and BWA was used to map the clean reads to the reference genome of *Liriodendron*, which is available from: https://ftp.cngb.org/pub/CNSA/data2/CNP0000815/CNS0044063/CNA0007303. GATK was used with standard filtering parameters (https://gatk.broadinstitute.org) to call and filter SNP variants^[25]^. VCFTOOLS^[26]^ was used to control the genotype data, removing SNP loci with a detection rate of less than 0.01, mass ≤ 30, minimum allele frequency less than 0.05, and minimum sequencing depth greater than 3 per SNP, and retaining only bi-alleles (i.e., AA, coded as 0; Aa, coded with 1; AA coded as 2). Ultimately, 192,972 informative SNPs were retained, and BEAGLE^[27]^ was used to further estimate missing genotypes for subsequent GWAS analysis and annotate the SNPs using ANNOVAR^[28]^. The quality control conditions for InDels were QualBy Depth (QD) < 2.0, RMS Mapping Quality (MQ) < 40.0, Fisher Strand (FS) > 200.0, Strand Odds Ratio (SOR) > 10.0, Mapping Quality Rank Sum Test (MQ Rank Sum) < −12.5, Read Pos Rank Sum Test (Read Pos Rank Sum) < −20, and removed individuals with deletion rates of more than 5%, resulting in the retention of 60,666 InDel loci and BEAGLE was used to further estimate the missing genotypes.

### Measurement of phenotypic traits

In September 2023, we collected the phenotypic data of 233 sample trees. The growth traits measured included clear bole height (CBH), crown length ratio (CLR), tree height (H), and diameter at breast height (DBH). CBH and H were measured using an ultrasonic altimeter, while DBH was measured at 1.3 m of tree height using a tape measure, and CLR was calculated as the ratio of crown length to tree height. The stem form traits measured included straightness (ST), forking (FK), number of branches (NB), and branching angle (BA). NB was determined by visually counting all first-order branches of individual trees, and the other three traits were measured using grading scores (Supplementary Table S1). All phenotypic data were subsequently summarized (Supplementary Table S2). Descriptive statistics for the phenotypic data included mean, maximum (max), minimum (min), standard deviation (SD), and coefficient of variation (CV).

### Genome-wide association analysis

Principal component analysis was conducted on the filtered InDel data using the GAPIT package^[29]^, and the first three principal components were used to plot 3D scatter plots in R using the SCATTERPLOT3D package. Pritchard et al. corrected for spurious correlations by applying group structure (Q) estimated using a set of random markers to a general linear model analysis^[30]^. The equations for the general linear model (GLM) used in GWAS was as follows:

1 $\rm {y} = {X\beta } + {Qv} + {e} $

The equation for the mixed linear model (MLM) used in GWAS was as follows^[31]^:

2 $ \mathrm{y} = {X\beta } + {Qv} + {Zu} + {e} $

where y represents the vector of observed phenotypes, X represents a matrix of molecular markers, β represents an unknown vector of additive SNP effects as fixed effects, v represents a vector of population structure fixed effects, u represents a multigene vector of kinship backgrounds as a random effect, Q and Z are their incidence matrices, and e represents a random residual error. Using the GAPIT package in R, the kinship matrix and the first three principal components in the principal component analysis were used as covariates in the mixed linear model. In addition to these two models, we also employed CMLM, MLMM, BLINK, and FarmCPU models to identify significant loci (−Log_10_(P) ≥ 4.50). The association effects were compared, and the appropriate models were ultimately selected for association analyses of the different traits. The R package ggplot was used to generate Manhattan and quantile-quantile plots (QQ plots).

### Identification and functional annotation of candidate genes

Candidate genes were identified within a 20.0 kb region upstream and downstream of the significant SNP site. The protein sequences of the candidate genes were obtained from the TAIR website (www.arabidopsis.org), and their functions were inferred by comparing the protein sequences of homologous genes in *Arabidopsis thaliana*, filtering the results to select those with the highest matches.

### Expression assay of candidate genes

In 2019, we selected young spring leaves, stem tips during the twitching period, and summer-differentiated xylem as material. Leaf blades were sampled in late March 2019 from the secondary branches of the first live branch, at the position of the 2^nd^ to 3^rd^ leaves near the base of the branch, in both the southeast and northwest directions. Stem tips were collected in late April 2019 from the top buds of secondary branches of the first live branch in the southeast and northwest directions during the peak twitching season. In mid-July 2019, the surface active primary xylem cells were scraped at breast height after peeling the bark in the southeast direction. These were quickly cooled in liquid nitrogen, transported back to the laboratory, and stored in a −80 °C freezer for subsequent transcriptome sequencing. All samples included three tissues/organs from three parents, each with two biological replicates; and two F1 full-sib families, with eight progenies from each family, resulting in 66 total samples for transcriptome sequencing. Subsequently, a differentially expressed gene (DEG) set was obtained, which was obtained by comparing the differences in gene expression between the dominant cross combination (MSL × WYS) and the non-dominant cross combination (MSL × S) and filtering out the genes with a *p*-value < 0.05 and log_2_FC > 1. The two cross combinations showed significant differences in growth, so the candidate genes identified in this study were cross-referenced with the DEG set to preliminarily validate the candidate genes selected from association analysis.

## Results

### Statistical analysis of phenotypic data

**Figure 1:**
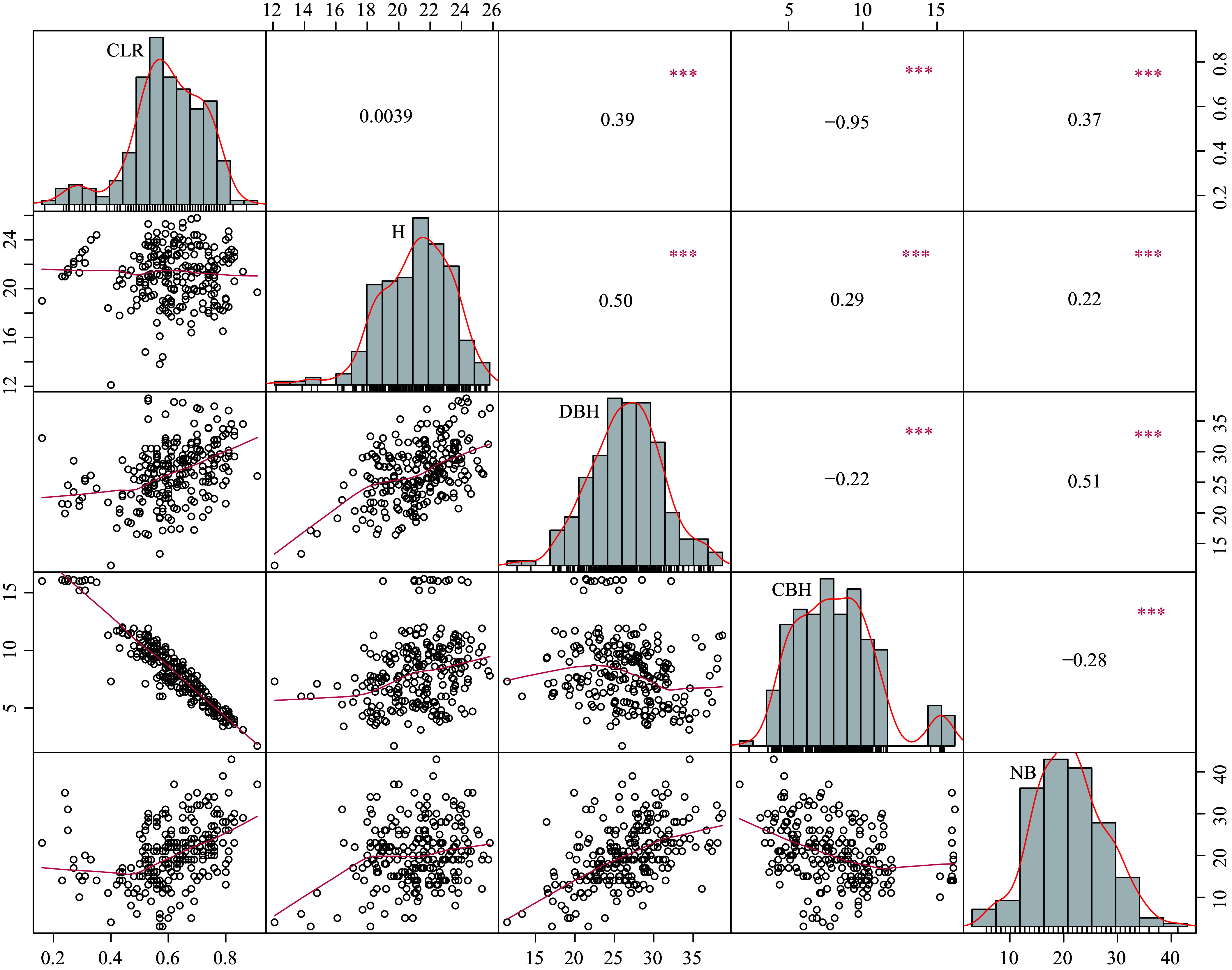
Scatterplot matrix, density distribution plot, and correlation coefficients (Pearson's r) for quantitative traits (tree height (H), diameter at breast height (DBH), number of branches (NB), clear bole height (CBH), crown length ratio (CLR)). * 0.01 < *p* < 0.05; ** 0.001 < *p* < 0.01; ***p < 0.001.

Descriptive analyses for five quantitative traits was conducted: H, DBH, NB, CBH, and CLR (Table 1). The results showed that H ranged from 12.10 to 25.80 m, DBH ranged from 11.40 to 38.70 cm, NB ranged from 3 to 43, CBH ranged from 1.70 to 16.20 m, and CLR ranged from 0.16 to 0.91. The coefficients of variation for the five traits ranged from 11% (H) to 37% (CBH), indicating significant differences among individuals. We plotted frequency histograms for the three graded traits of ST, FK, and BA (Supplementary Fig. S1) to better visualize the structure of the data. Subsequently, we performed normality tests for the five quantitative traits and found that they generally conformed to a normal distribution (Fig. 1), allowing for subsequent GWAS. The results of phenotypic correlation analyses of quantitative traits showed that there was a strong negative correlation between CBH and CLR, a moderate positive correlation between DBH and CLR, as well as NB, and a weak positive correlation between CLR and DBH and NB.

**Table 1:** Statistical analysis of tree height (H), diameter at breast height (DBH), number of branches (NB), clear bole height (CBH), crown length ratio (CLR) traits.

Traits	Minimum values	Maximum value	Average value	Standard deviation	CV (%)
H	12.10	25.80	21.21	2.28	0.11
DBH	11.40	38.70	26.64	4.91	0.18
NB	3.00	43.00	20.40	7.29	0.36
CBH	1.70	16.20	8.15	3.00	0.37
CLR	0.16	0.91	0.62	0.13	0.21

### Resequencing and genetic loci detection

**Figure 2:**
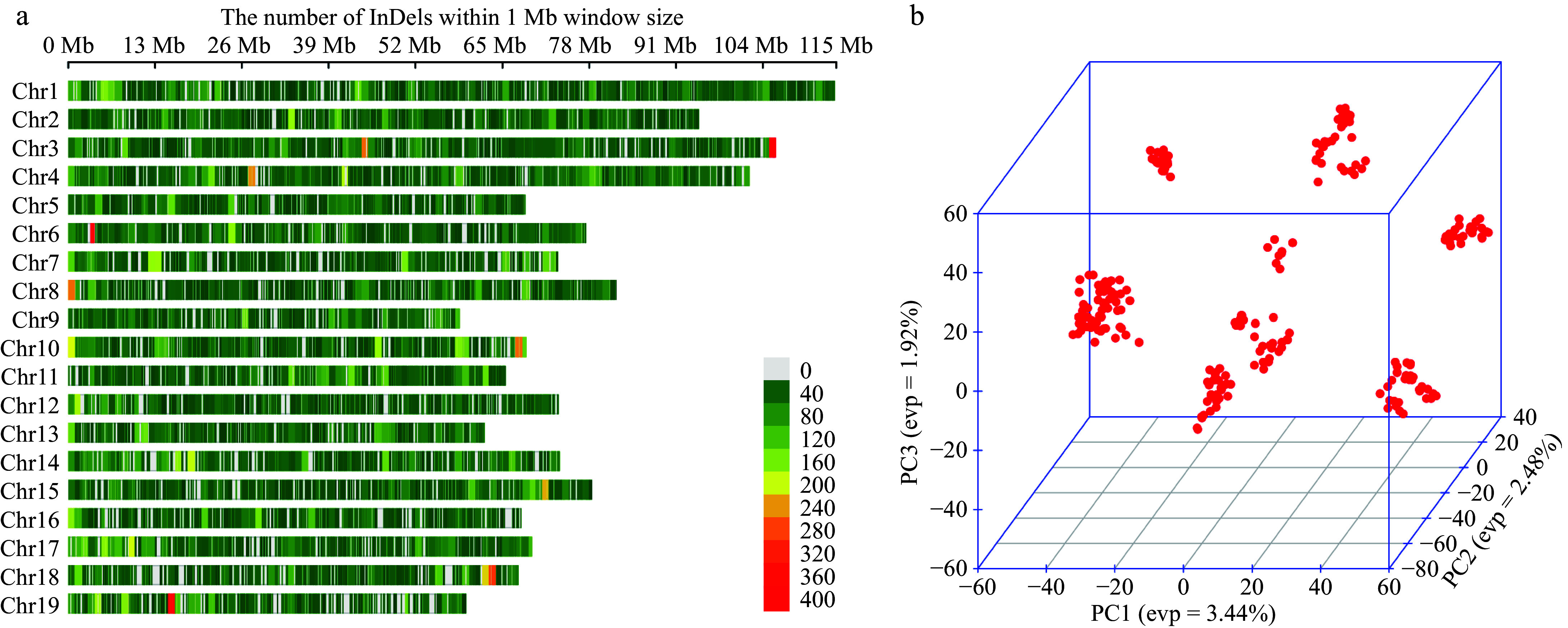
(a) InDel density map of 19 chromosomes, with a window size of 1 MB, excluding overlapping clusters, where redder colours represent denser InDel distributions. (b) Three-dimensional scatter plot of the first three principal components for all individuals.

Regarding the detection of SNPs, there was a detailed elaboration in our previous study^[19]^. All our selected SNPs, can be classified into six types of nucleotide substitutions (Supplementary Fig. S2). The C to T (T to C) type has the highest percentage of 50.68% and the G to C (C to G) type has the lowest percentage of 4.42%. The transition ratio (Ti) and transversion ratio (Tv) can assess the quality of SNPs in the genome^[32]^. In general, the ratio of Ti/Tv does not exceed 4, and a higher ratio generally represents a better quality of sequencing^[33]^. In this study, the Ti/Tv ratio of SNPs was 2.97, suggesting that the SNPs had good quality. After quality control, we obtained a total of 60,666 InDel loci, evenly distributed on 19 chromosomes (Fig. 2a). The results of principal component analysis showed (Fig. 2b) that the first three principal components accounted for a total of 9.32%, and we used the first three principal components to plot a three-dimensional scatter plot, revealing that individuals could be classified into distinct subgroups, suggesting the presence of population structure.

### Selection of GWAS models

**Figure 3:**
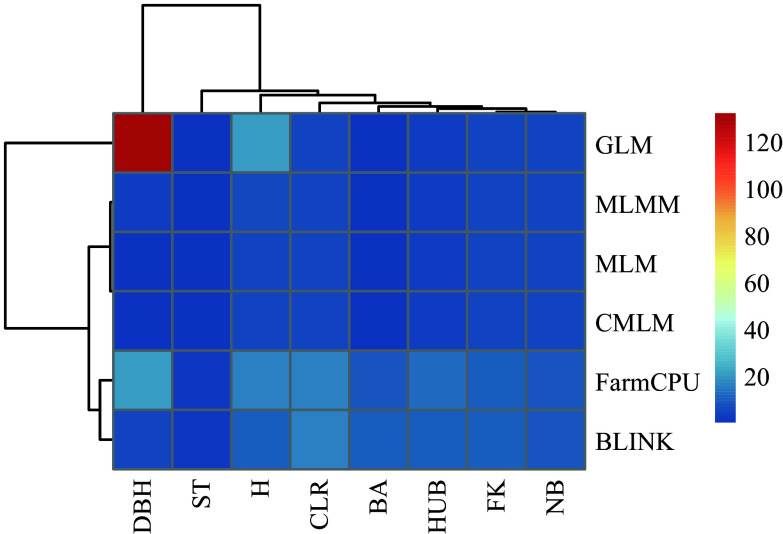
Heat map of the number of significant loci screened by each model. The redder the color, the higher the number of loci.

**Figure 4:**
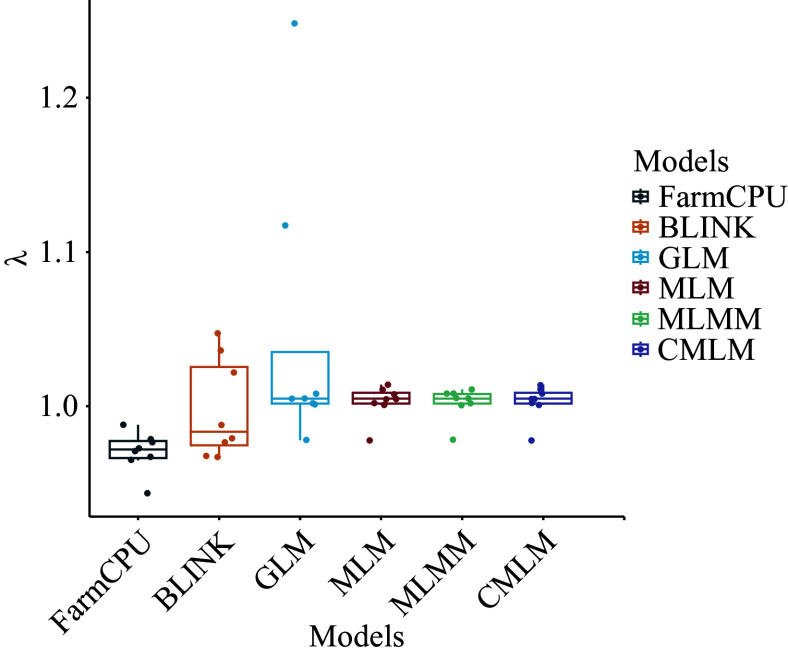
Box plots of expansion coefficients for the six models. λ is the expansion coefficient.

In this study, we compared the results of six association models: GLM, MLM, CMLM, MLMM, FarmCPU, and BLINK, and selected the most suitable model for association analysis. This approach made the association results more accurate and reliable. For SNP-based association analysis, we counted the number of significant loci identified by each model and plotted a heat map after normalizing the data (Fig. 3). Additionally, we calculated the expansion coefficients (λ) for each model and visualized them with box plots for a more intuitive comparison (Fig. 4). For example, in the case of the DBH trait, the GLM model identified the most significant loci, with a total of 133, while the CMLM model identified the least, with only one. However, the expansion coefficient (λ) for the GLM model was 1.248, suggesting the possibility of false-positive results, which could compromise accuracy. In contrast, the CMLM model may have been overcorrected, leading to a false-negative situation, which resulted in fewer significant loci being identified. Overall, the MLM, CMLM, and MLMM models screened fewer significant loci, although their λ values were close to 1.00. The GLM model detected more significant loci but had greater fluctuation in its λ values. After evaluating the significant loci identified by each model and their accuracy, we decided to use the FarmCPU and BLINK models for subsequent association analysis.

### Genome-wide association analysis and candidate gene screening

**Figure 5:**
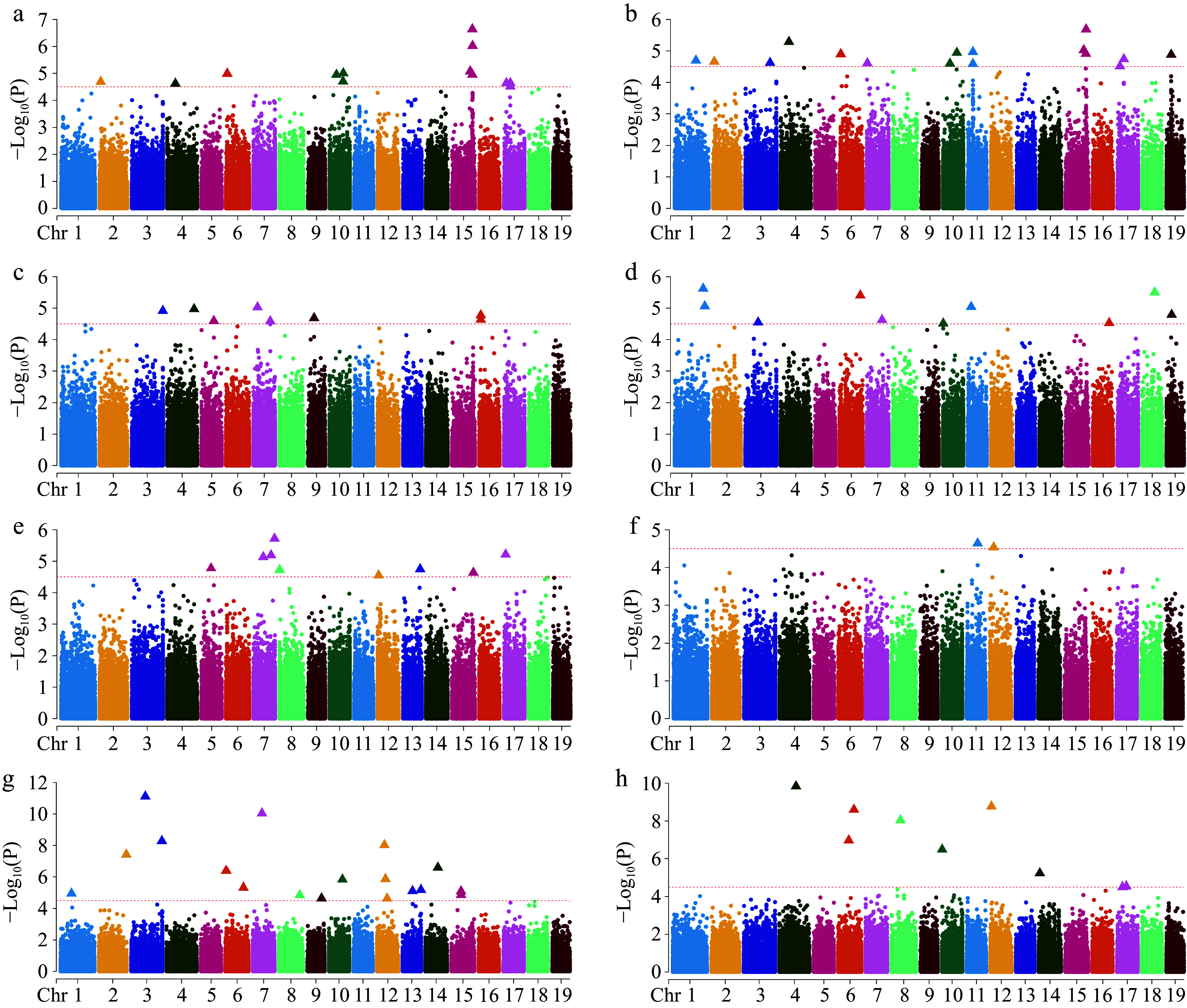
Manhattan plot of GWAS analysis using SNPs. (a) Clear bole height; (b) crown to length ratio; (c) branch angle; (d) forking; (e) number of branches; (f) straightness; (g) diameter at breast height; (h) tree height. The red dotted line indicates the significance threshold (−Log_10_(P) ≥ 4.50), the points of the triangle are the loci of significance, and only 19 chromosomes are shown, excluding contigs.

In this study, we analyzed the association of eight traits and visualized Manhattan and QQ plots (Fig. 5 & Supplementary Fig. S3). We identified a total of 155 significant loci, including 97 SNP loci and 58 InDel loci. Among the screened significant SNP loci, 18 SNPs were significantly correlated with CLR, 14 SNPs were significantly associated with CBH, three SNPs were significantly correlated with ST, 11 SNPs were significantly correlated with FK, nine SNPs were significantly correlated with BA, 10 SNPs were significantly correlated with the NB, 21 SNPs were significantly correlated with DBH, and 11 SNPs were significantly correlated with H. A total of 87 candidate genes were screened among the 97 significant SNPs (Supplementary Table S2), with more genes associated with growth traits (22 for CLR, 18 for CBH, 10 for H, and nine for DBH) than with stem form traits (two for ST, five for FK, 10 for BA, and 11 for NB). Using the significance SNP loci, we screened for multiple common genes in the traits of CLR and CBH, such as *Lchi_15g29601, Lchi_15g29602, Lchi_15g29603, Lchi_2g03172, Lchi_15g29902, Lchi_15g29903, Lchi_15g29904*, etc., which may suggest that a single gene can influence multiple traits. We screened a total of 74 candidate genes in the 58 significant InDel loci (Supplementary Table S3), of which the most candidate genes were associated with the traits of DBH and BA, both of which were 19; and the least candidate genes were associated with the traits of CBH and NB, which were only two. We identified three common genes shared by both SNP and InDel significance loci: *Lchi_15g29902, Lchi_15g29903,* and *Lchi_5g12151*. The two candidate genes *Lchi_15g29902* and *Lchi_15g29903* were associated with both CLR and CBH, and the *Lchi_5g12151* candidate gene was associated with BA.

**Figure 6:**
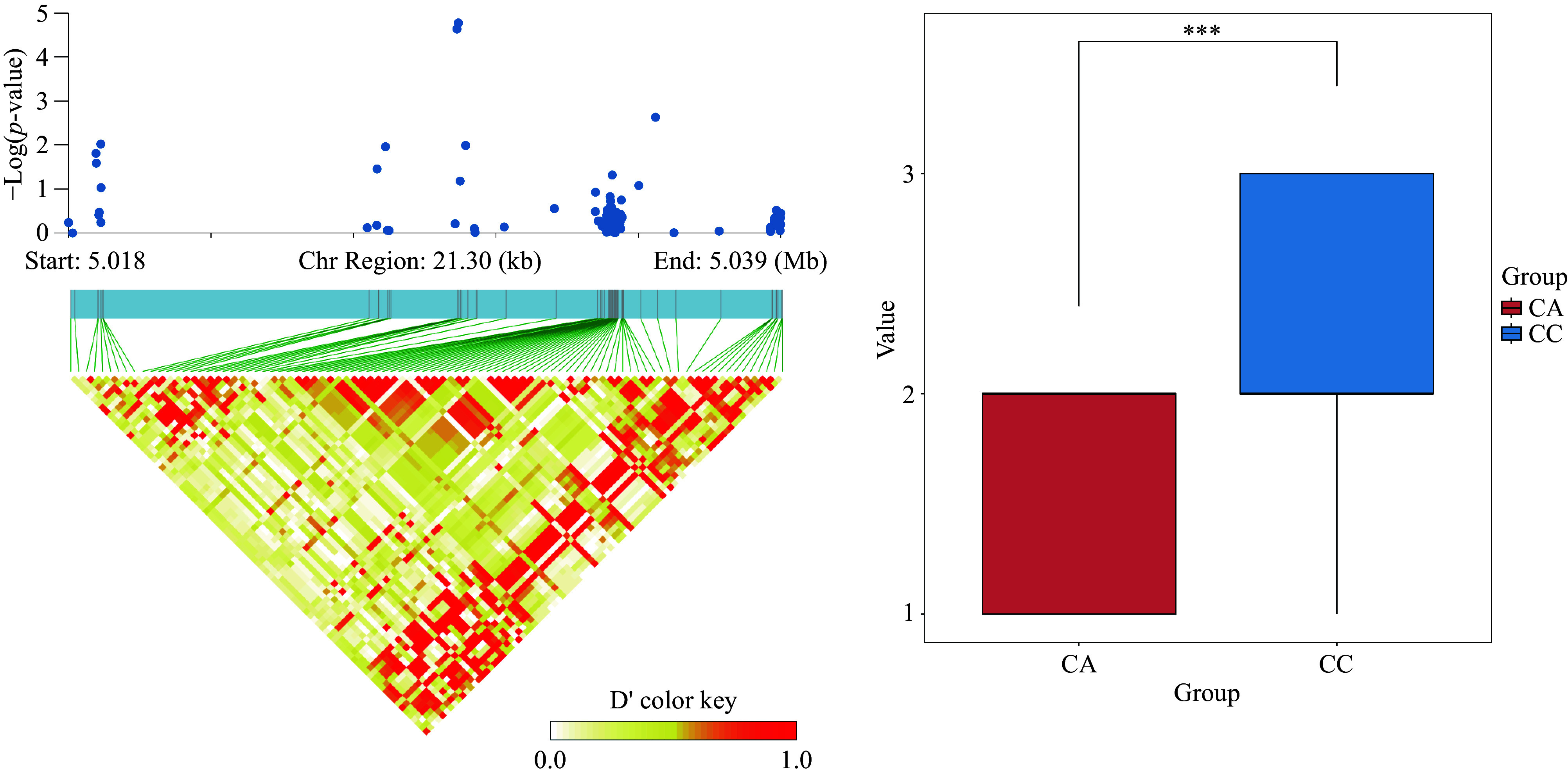
(a) Analysis of significant SNP locus Chr16_5029808 LD block. (b) Significance analysis of allelic differences for the highly significant SNP locus chr16_5029808, where CC indicates that the locus is CC pure and CA indicates that the locus is CA heterozygous.

Candidate genes were selected from our previously updated gene set^[34]^. Following the screening process, we identified the candidate gene *Lchi_16g30522* on chromosome 16, associated with the branching angle trait. Haplotype analysis indicated that this SNP locus is tightly linked (Fig. 6). The gene was identified through a genetic analysis on the TAIR website (www.arabidopsis.org) by comparing protein sequences. We found that this gene shows high similarity to OPT3 in *A. thaliana*, which encodes a phloem-specific iron transport protein that loads iron into the phloem, facilitates iron recirculation from xylem to phloem, and regulates iron signaling from shoots to roots and iron redistribution from mature to developing tissues. A recent report indicates that OPT3 also transports Cu in *A. thaliana*^[35]^. Fe and Cu play a significant role in plant growth and development and are essential trace elements in plants. These two elements can affect the photosynthesis and respiration of plants^[36,37]^. Recent studies have futher demonstrated that Cu deficiency alters the branching structure of plants^[38]^.

The candidate gene *Lchi_2g03172,* was identified on chromosome 2 for its association with crown length ratio traits. After comparison, it was found to be highly homologous to chloroplast fructose-1,6-bisphosphate aldolase (FBA3) in *A. thaliana*, which plays a role in the regeneration phase of the Calvin cycle^[39]^. Grafting experiments in *A. thaliana* demonstrated that FBA3 loses its function during leaf phloem translocation, leading to the accumulation of photosynthetic products in leaves and growth retardation^[40]^. Additionly, we identified the candidate gene *Lchi_10g19986* on chromosome 10, which is highly homologous to *AtNug2* in *A. thaliana.* *AtNug2* is involved in the maturation process of ribosomal subunit before the 60S stage in plants and is more strongly expressed in meristematic tissues. Mutations in *AtNug2* can cause growth retardation in *A. thaliana*^[41]^.

### Expression of candidate genes in shoot apices and xylem

Differences in gene expression can effectively explain phenotypic variation, and the association between genes and traits can be better understood by analyzing the expression of candidate genes across different tissues. From the candidate genes screened using SNP and InDel loci, we identified nine candidate genes in the DEG collection of two heterozygous combinations with significant differences in growth traits (Table 2). These genes were primarily expressed in the stem tip and xylem. The up-regulated genes included *Lchi_12g23550*, *Lchi_18g34878*, *Lchi_12g23559*, *Lchi_15g28864*, and *Lchi_14g27994*, while the down-regulated genes were *Lchi_10g20626*, *Lchi_6g12913*, *Lchi_5g12415,* and *Lchi_3g07857*. The genes *Lchi_18g34878* and *Lchi_10g20626* were associated with DBH, while the *Lchi_12g23550* gene was associated with H. In addition, *Lchi_14g27994* and *Lchi_3g07857* were associated with FK and ST, respectively. These findings suggest that differential expression of candidate genes may be associated with growth and stem form traits.

**Table 2:** Nine candidate genes associated with growth and stem traits in the differentially expressed gene (DEG) sets.

Tissues	Gene	Log_2_FC	Log_2_CPM	LR	*p*-value	FDR	Up/down
Shoot	*Lchi_12g23550*	2.45	3.12	20.99	4.63E-06	3.78E-04	Up
	*Lchi_18g34878*	3.61	2.75	13.81	2.02E-04	6.66E-03	Up
	*Lchi_10g20626*	−1.53	5.14	14.37	1.51E-04	5.34E-03	Down
	*Lchi_12g23559*	1.98	4.25	8.97	2.75E-03	4.24E-02	Up
	*Lchi_5g12415*	−2.05	3.59	18.10	2.10E-05	1.18E-03	Down
	*Lchi_15g28864*	3.34	2.07	10.34	1.3E-03	2.51E-02	Up
	*Lchi_14g27994*	2.57	2.35	11.15	8.40E-04	1.89E-02	Up
	*Lchi_3g07857*	−1.32	7.58	9.09	2.57E-03	4.07E-02	Down
Xylem	*Lchi_6g12913*	−1.06	6.54	15.94	6.52E-05	3.14E-03	Down

## Discussion

GWAS, based on the fundamental principle of linkage disequilibrium (LD), is a comprehensive method for investigating the genetic architecture of natural populations^[42]^. SNPs are single-base mutation, and when a synonymous mutation occurs, it does not lead to amino acid changes due to codon redundancy, thereby maintaining protein stability. In contrast to SNP, InDel involves multi-base insertions or deletions, and when it occurs in the CDS region, it is more likely to cause transcription and translation errors or premature termination, leading to phenotypic changes. Studies have shown that InDels have a greater impact on protein structure and function than SNP^[43]^. InDels can alter protein conformation, affecting key traits of mitochondrial genes^[44]^. Therefore, in this study, GWAS utilizing both SNP and InDel loci enabled more comprehensive screening of significant loci and accelerated the genetic improvement process. Among the nine differentially expressed genes identified, *Lchi_6g12913* was associated with the significant SNP locus Chr_3590886, where a non-synonymous mutation (A to T) was detected, potentially altering the amino acid sequence. This gene showed differential expression in the two-hybrid combinations.

The statistical models utilized in GWAS have evolved to achieve greater completeness and efficiency. These models are primarily based on the general linear model (GLM) and mixed linear model (MLM). GLM incorporates Principal Component Analysis (PCA) or Population Structure (Q) as covariates to enhance calculation accuracy. Conversely, the MLM integrates a Kinship (K) matrix as a covariate to mitigate false positives in association analyses^[45]^. The CMLM model corrects for loci filtered out due to overcorrection in the MLM model and calculates the average kinship after clustering and grouping kinship data, significantly improving computational efficiency^[46]^. MLMM is a stepwise regression method based on the most significant loci for phenotype-genotype association analyses. The most significant loci are entered as covariates in the next step of the calculation, and the loci that are significantly associated with the phenotype are obtained step by step^[47]^. As sequencing data volume increases, computational speed becomes a major problem. The FarmCPU model improves computational speed and accuracy, performing association analysis more efficiently by using possible associated loci as covariates for fixed effects, then calculating associations of loci through random effects. The final results are output when the two effects alternate until no new associated loci appear. In our study, we observed differential model performance across traits when using SNPs for association analysis. Notably, the BLINK model performed best in associating the tree height trait (Supplementary Figs S4−S11), while the FarmCPU model outperformed others across different traits. Additionally, when employing InDel loci for correlation analysis, the Blink model exhibited enhanced correlation efficacy. However, for the diameter at breast height trait, the FarmCPU model outperformed the BLINK model, identifying more significant loci and showing more pronounced deviations in the QQ plot (Supplementary Figs S12−S19). These observations highlight that model selection is critical for ensuring the accuracy and reliability of association analyses, with each model demonstrating specific strengths and limitations across traits and genetic variants.

Plants require a variety of trace elements for optimal growth and development, with iron and copper being essential. These elements trigger the transcription of genes involved in iron uptake and transport, mitigating iron deficiency^[48]^. Plants also tightly regulate iron metabolism to prevent toxicity from iron overload^[35]^. In *A. thaliana*, OPT3 is identified as a phloem-specific iron transport protein^[49]^. It plays a key role in signaling iron demand from shoots to roots and facilitates iron transport. Recent findings suggest that OPT3 is involved in maintaining copper homeostasis, allowing plants to regulate the transport of both elements to support branch development and avoid toxicity^[35]^. Moreover, copper exerts a significant influence on *A. thaliana* branch architecture and the fertility of male *A. thaliana*^[38]^. In the present study, the candidate gene *Lchi_16g30522* was examined, which exhibits high homology to OPT3. *Lchi_16g30522* is likely to influence the growth, development, and branch structure formation in hybrid *Liriodendron* by regulating iron and copper levels. Through its homology with OPT3, *Lchi_16g30522* is a potential regulator of iron and copper homeostasis, thereby exerting significant effects on *Liriodendron* physiology.

Glycolysis is essential for converting glucose into pyruvate, with fructose-1,6-bisphosphate aldolase (EC 4.1.2.13, FBA) being a key enzyme in plants^[50]^. FBA is involved in various physiological and biochemical processes, such as plant growth, development, and response to drought stress^[51,52]^. Studies have shown that reduced FBA activity slows tomato growth^[53]^. In moso bamboo, cFBA has been identified as a significant determinant, showing higher activity in elongating tissues than in those that have completed elongation^[54]^. Alterations in FBA activity in potatoes significantly affect photosynthesis and carbon allocation, underscoring the crucial role of FBA in potato growth^[55]^. In this study, we identified the candidate gene *Lchi_2g03172* as highly homologous to chloroplast fructose-1,6-bisphosphate aldolase (FBA3) in *A. thaliana*, making it an intriguing prospect. Previous studies in *A. thaliana* revealed that the FBA3 mutant exhibits a significant biomass reduction compared to the wild type (WT)^[40]^. We hypothesize that *Lchi_2g03172* may regulate hybrid *Liriodendron* growth by influencing photosynthesis and potentially impacting its canopy structure.

We screened three common genes (*Lchi_15g29902*, *Lchi_15g29903*, and *Lchi_5g12151*), utilizing both SNP and InDel markers. We performed protein sequence alignment of these three candidate genes and showed that *Lchi_15g29903* and *Lchi_5g12151* were poorly aligned, whereas *Lchi_15g29902* was highly homologous to PTF2 and encodes a novel plant-specific TFIIB-related protein that can interact with TBP2 and bind DNA. Mutation of PTF2 would result in failure of pollen germination and disruption of embryogenesis. Interestingly, studies in *Arabidopsis* have shown that PTF2 is not only expressed in developing pollen but is also abundantly expressed in other tissues with active cell division and differentiation, including the embryo and shoot apical meristem. Compared with the wild type, pollen-rescued *ptf2-1* plants have more lateral buds, suggesting that PTF2 also plays an important role in plant growth^[56]^. We hypothesized that *Lchi_15g29902* might have an effect on growth and pollen germination in *Liriodendron*.

Due to the long growth cycle of forest trees and the limited benefits of genetic improvement, breeders aim to maximize improvement within a shorter time frame. In this study, a GWAS was conducted for growth and stem-form traits were performed using SNP and InDel loci. In future studies, phenotypic traits can be observed across multiple locations and over successive years, utilizing multi-year, multi-site data to minimize environmental interference. For the identified candidate genes, some suitable genes can first be selected for experimental validation in *A. thaliana*. If conditions allow, homologous transformation can be attempted in *Liriodendron* for species-specific validation.

## Conclusions

A GWAS of growth and stem form traits in hybrid *Liriodendron* was conducted using SNP and InDel data and identified 97 SNP and 58 InDel loci associated with growth and stem form traits, respectively. Further, we discerned a total of 161 candidate genes, among which, the *Lchi_2g03172* and *Lchi_10g19986* genes might be related to growth, while *Lchi_16g30522* gene may have an impact on both growth and branching. The present results provide robust genetic loci for marker-assisted breeding and are conducive to mining genes related to growth and stem form traits, thereby accelerating the genetic improvement process of complex traits such as growth and stem form in *Liriodendron* plants.

## SUPPLEMENTARY DATA

Supplementary data to this article can be found online.

## Data Availability

All data generated or analyzed during this study are included in this published article and its supplementary information files.
