# Supplementary material for: Identification of genetic loci for growth and stem form traits in hybrid Liriodendron via a genome-wide association study
Source: For Res (Fayettev). 2025 Jan 22;5:e001. doi: 10.48130/forres-0025-0001 (PMC11870303; doi:10.48130/forres-0025-0001)
Supplement: Supplementary file 1 — Supplementary data to this article can be found online. [file forres-0025-0001-S1.zip › 10.48130_forres-0025-0001-Suppl-FigureS1.pdf]

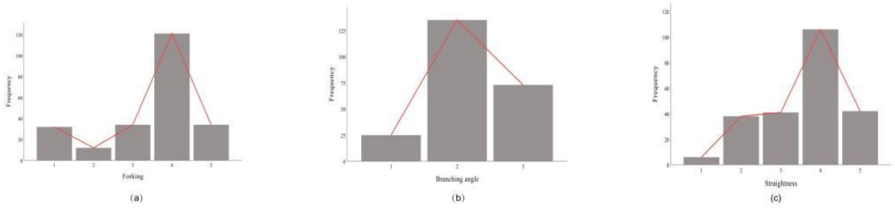


Fig. S1 (a) Frequency histograms for the forking trait; (b) frequency histograms for the branching angle trait; (c) frequency histograms for the straightness.
