# Supplementary figures and images for: Identification of genetic loci for growth and stem form traits in hybrid Liriodendron via a genome-wide association study
Source: For Res (Fayettev). 2025 Jan 22;5:e001. doi: 10.48130/forres-0025-0001 (PMC11870303; doi:10.48130/forres-0025-0001)

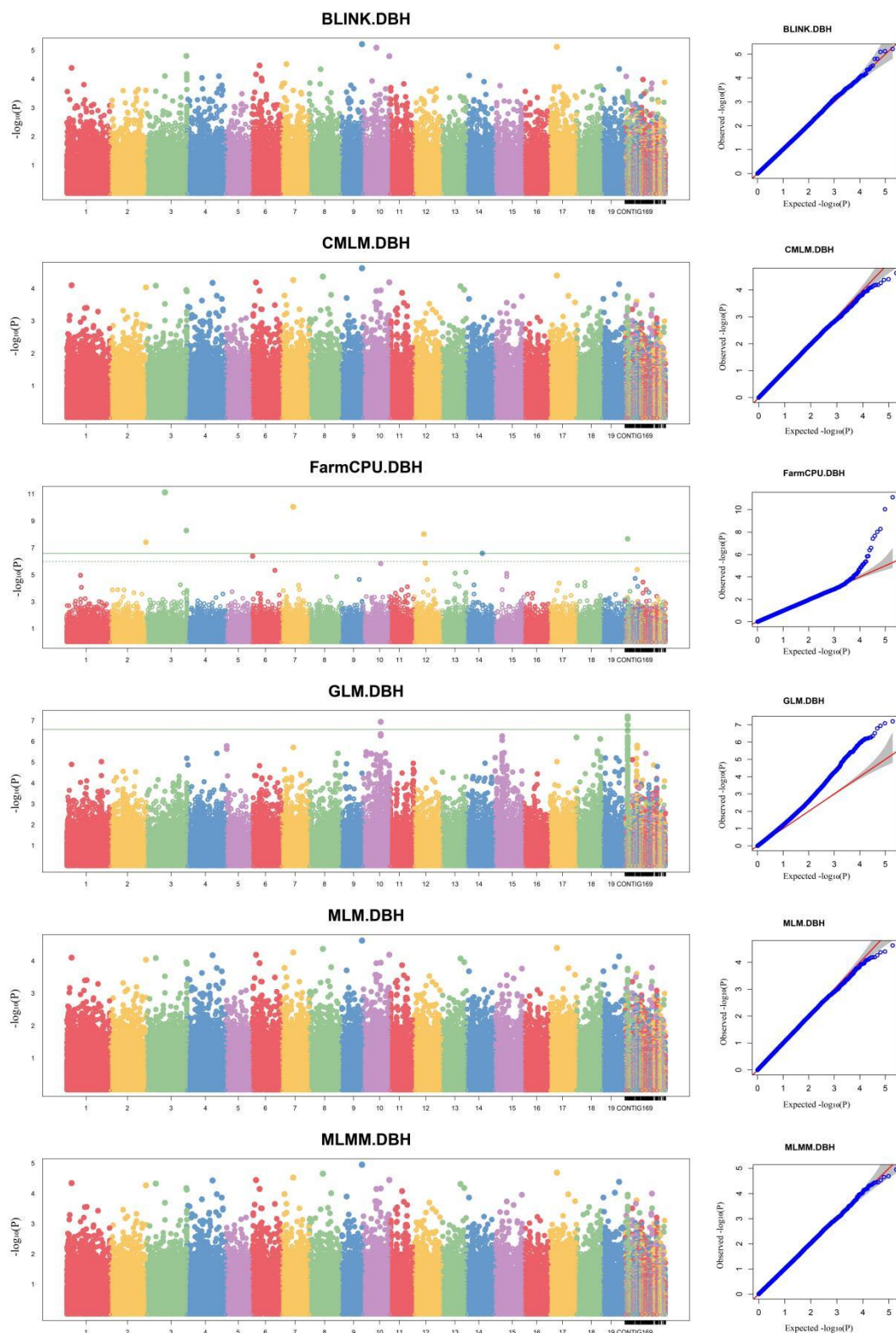

Fig. S10 Figure of the GWAS results for DBH using SNPs.

Supplement: Supplementary file 1 — Supplementary data to this article can be found online. [file forres-0025-0001-S1.zip › 10.48130_forres-0025-0001-Suppl-FigureS10.pdf]

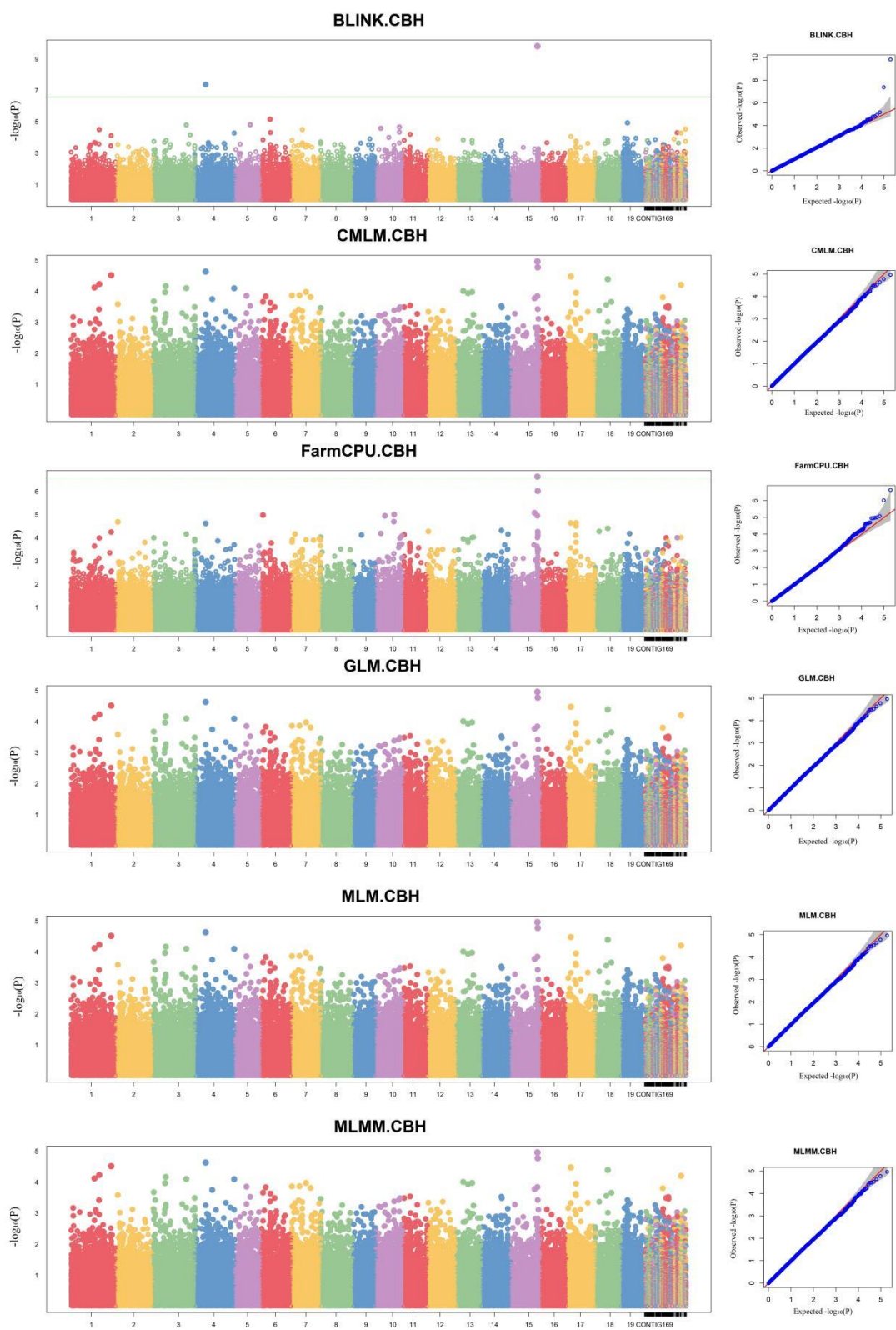

Fig. S11 Figure of the GWAS results for CBH using SNPs.

Supplement: Supplementary file 1 — Supplementary data to this article can be found online. [file forres-0025-0001-S1.zip › 10.48130_forres-0025-0001-Suppl-FigureS11.pdf]

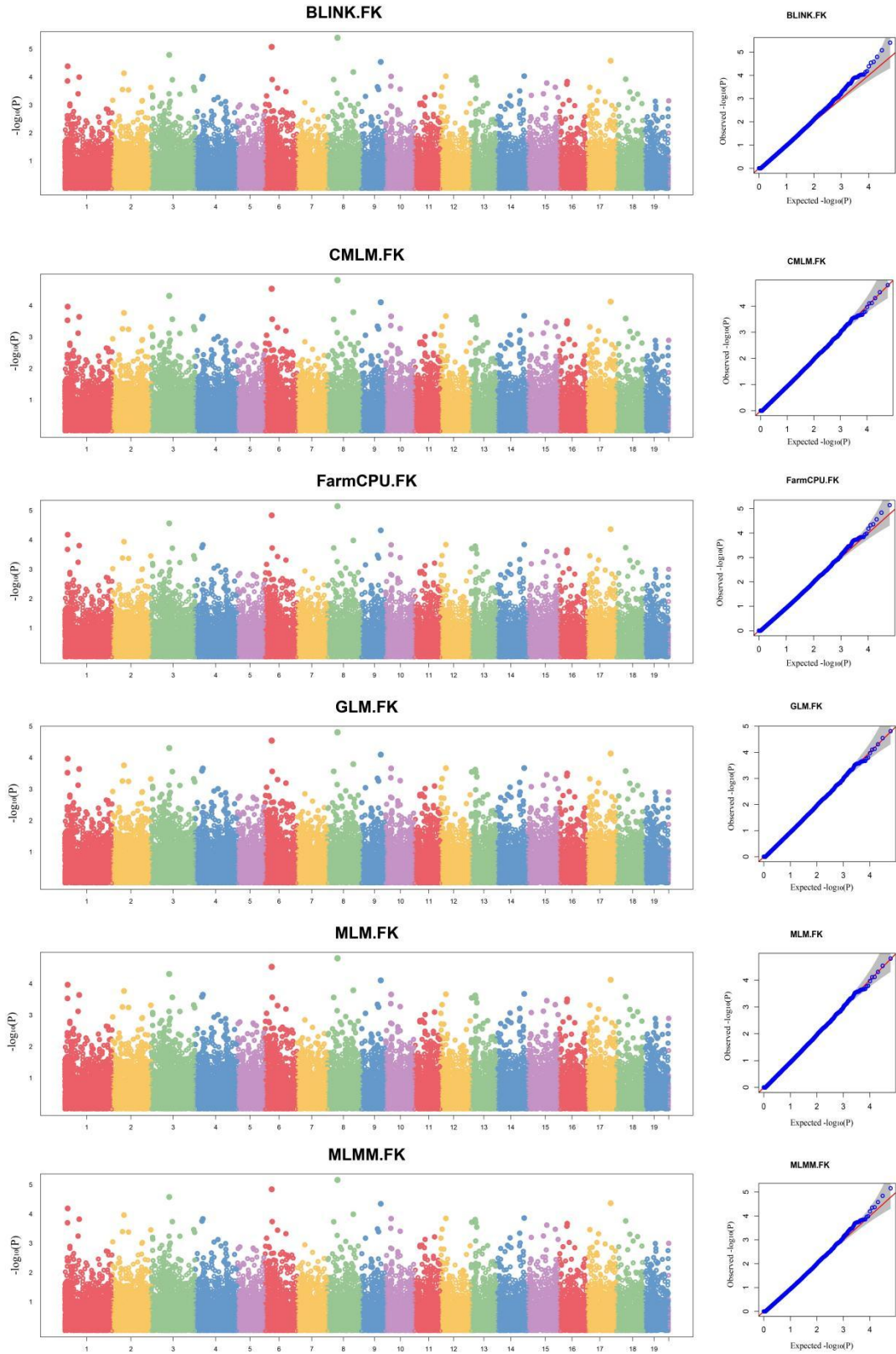

Fig. S12 Figure of the GWAS results for FK using InDels.

Supplement: Supplementary file 1 — Supplementary data to this article can be found online. [file forres-0025-0001-S1.zip › 10.48130_forres-0025-0001-Suppl-FigureS12.pdf]

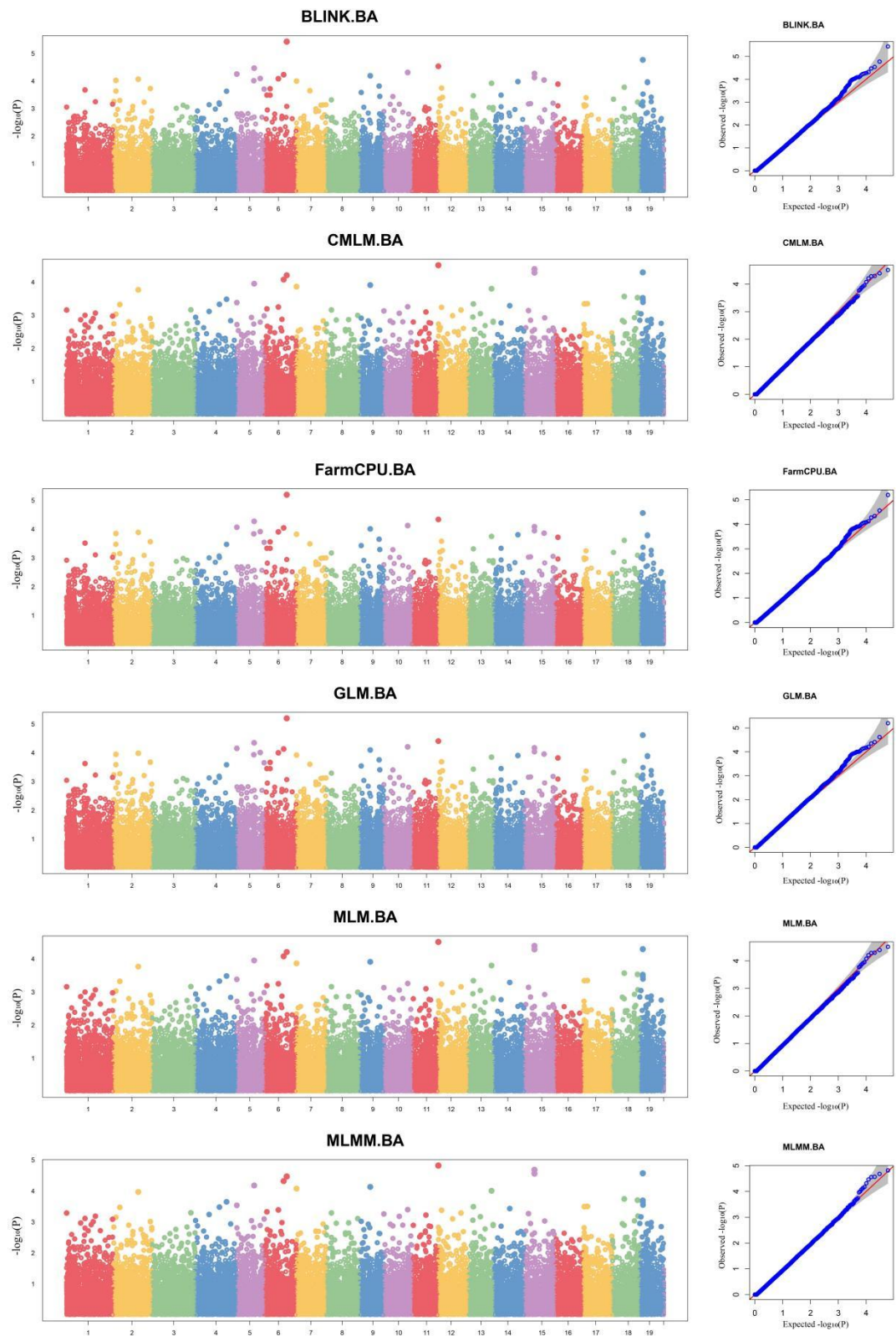

Fig. S13 Figure of the GWAS results for BA using InDels.

Supplement: Supplementary file 1 — Supplementary data to this article can be found online. [file forres-0025-0001-S1.zip › 10.48130_forres-0025-0001-Suppl-FigureS13.pdf]

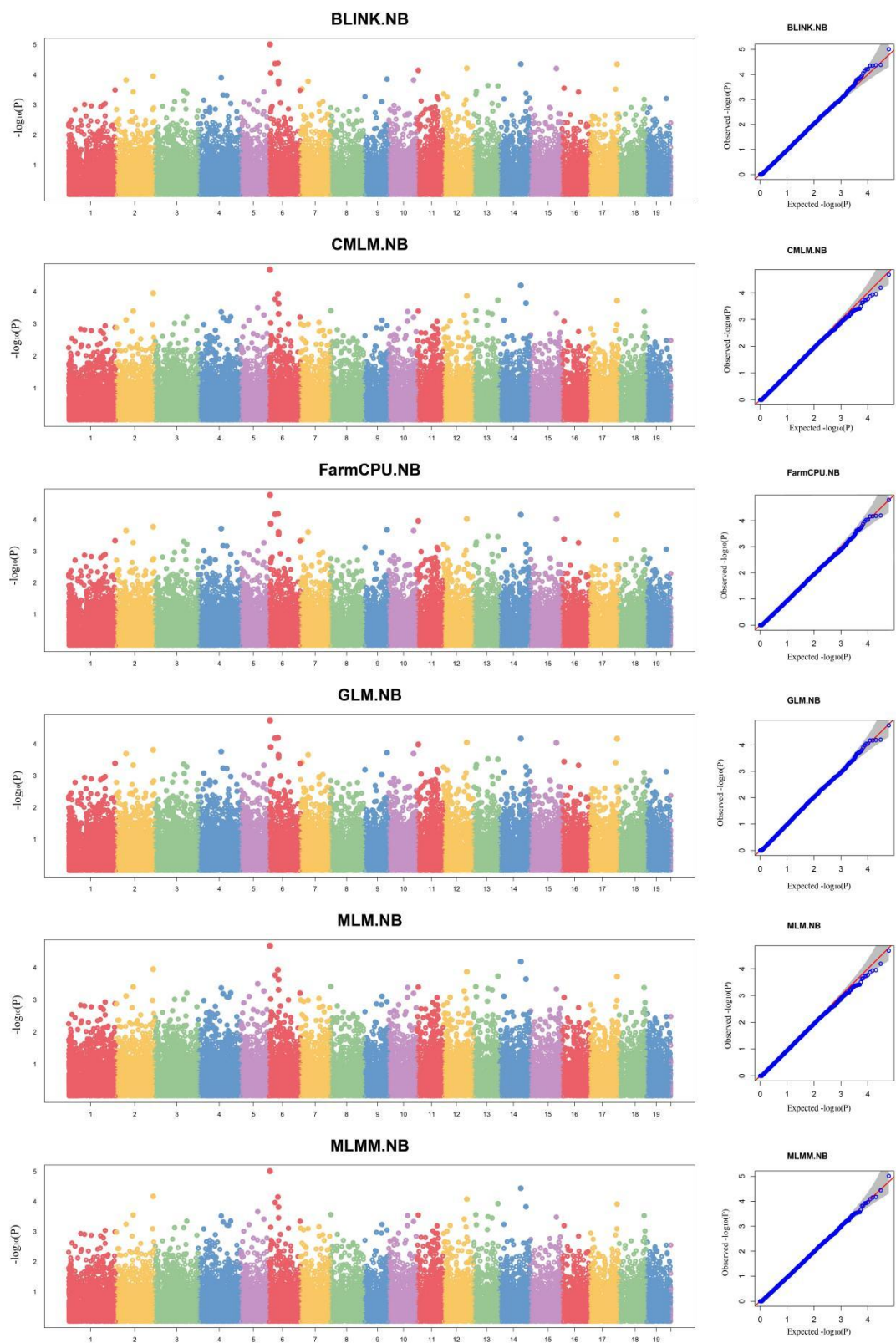

Fig. S14 Figure of the GWAS results for NB using InDels.

Supplement: Supplementary file 1 — Supplementary data to this article can be found online. [file forres-0025-0001-S1.zip › 10.48130_forres-0025-0001-Suppl-FigureS14.pdf]

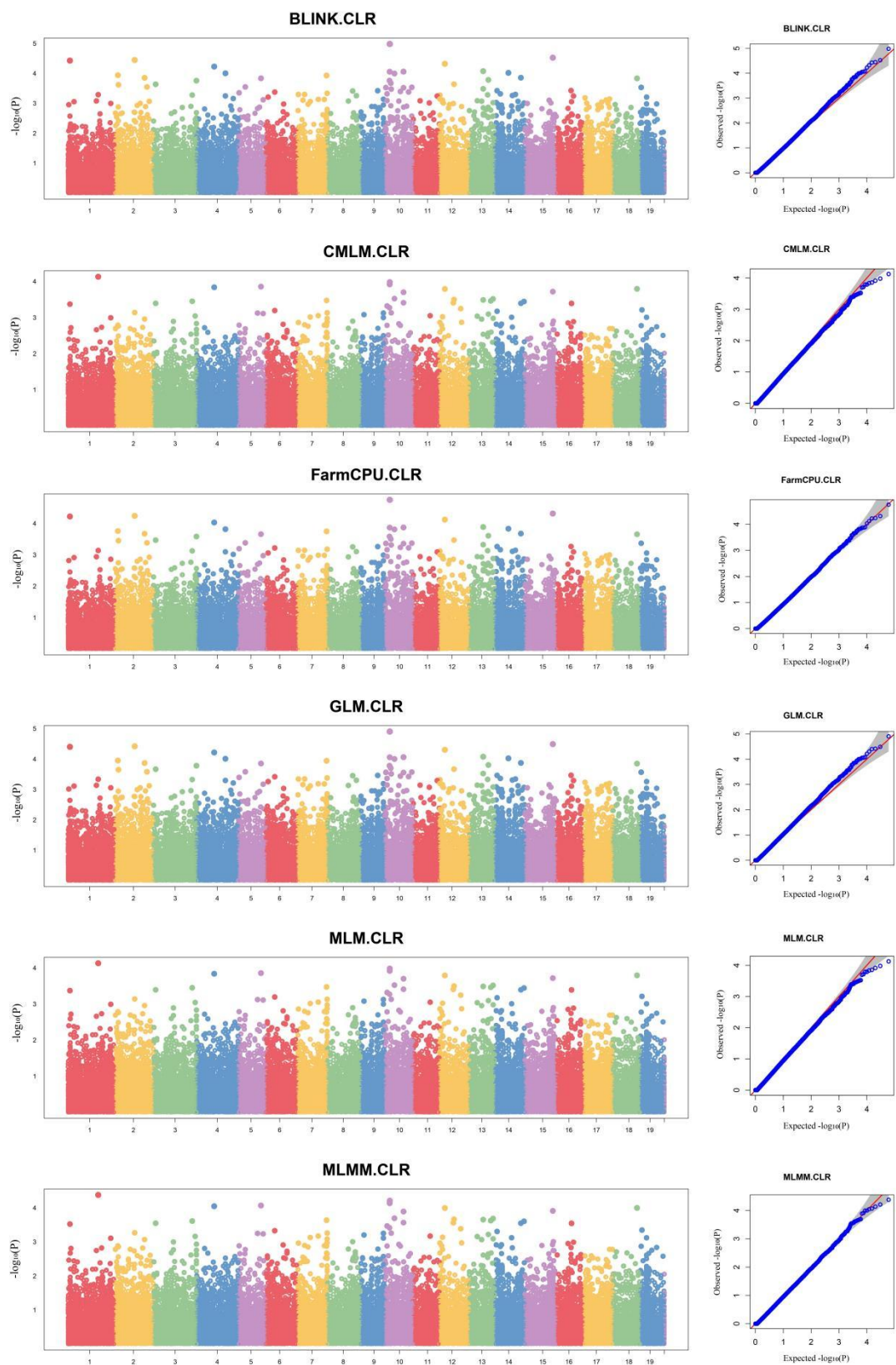

Fig. S15 Figure of the GWAS results for CLR using InDels.

Supplement: Supplementary file 1 — Supplementary data to this article can be found online. [file forres-0025-0001-S1.zip › 10.48130_forres-0025-0001-Suppl-FigureS15.pdf]

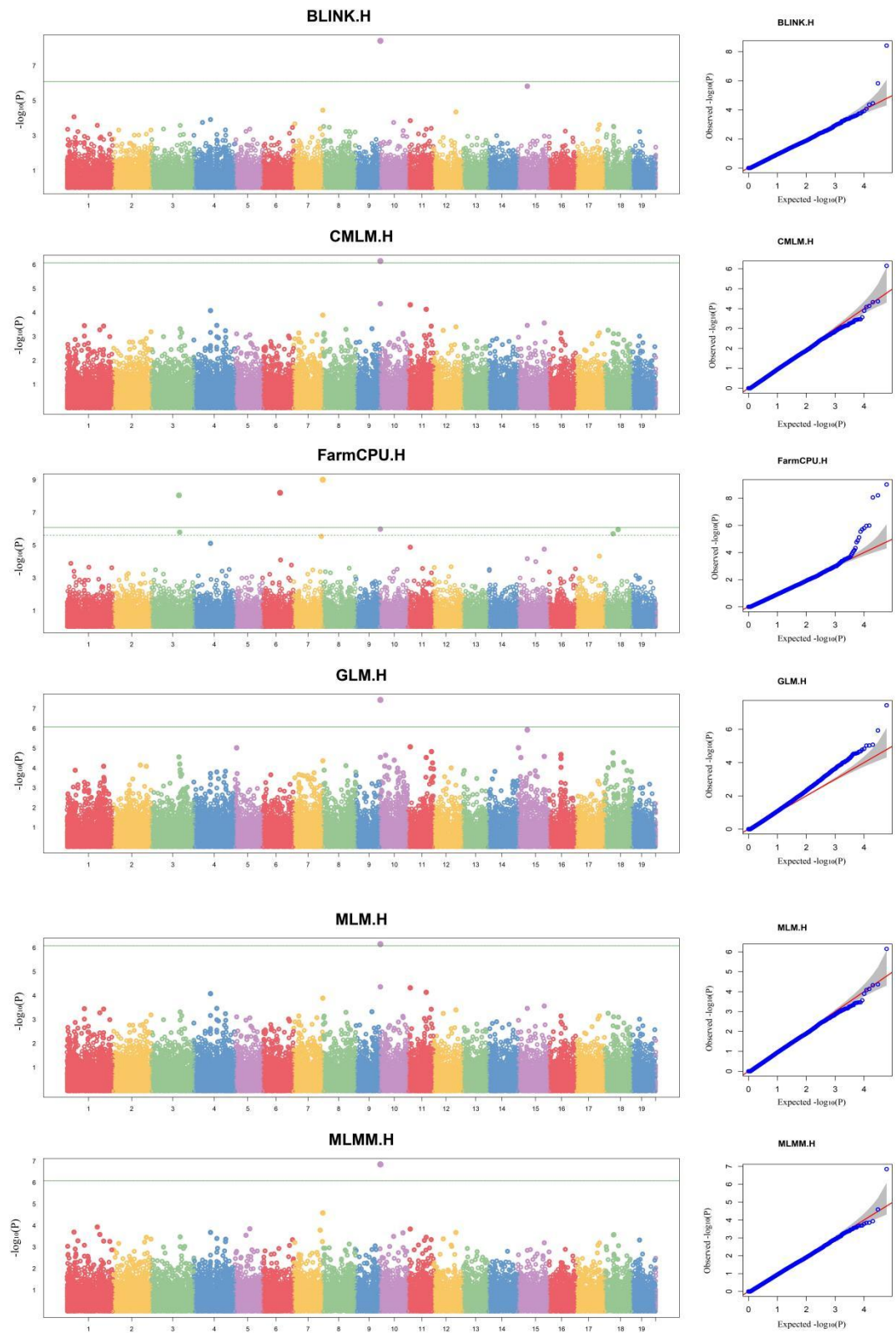

Fig. S16 Figure of the GWAS results for H using InDels.

Supplement: Supplementary file 1 — Supplementary data to this article can be found online. [file forres-0025-0001-S1.zip › 10.48130_forres-0025-0001-Suppl-FigureS16.pdf]

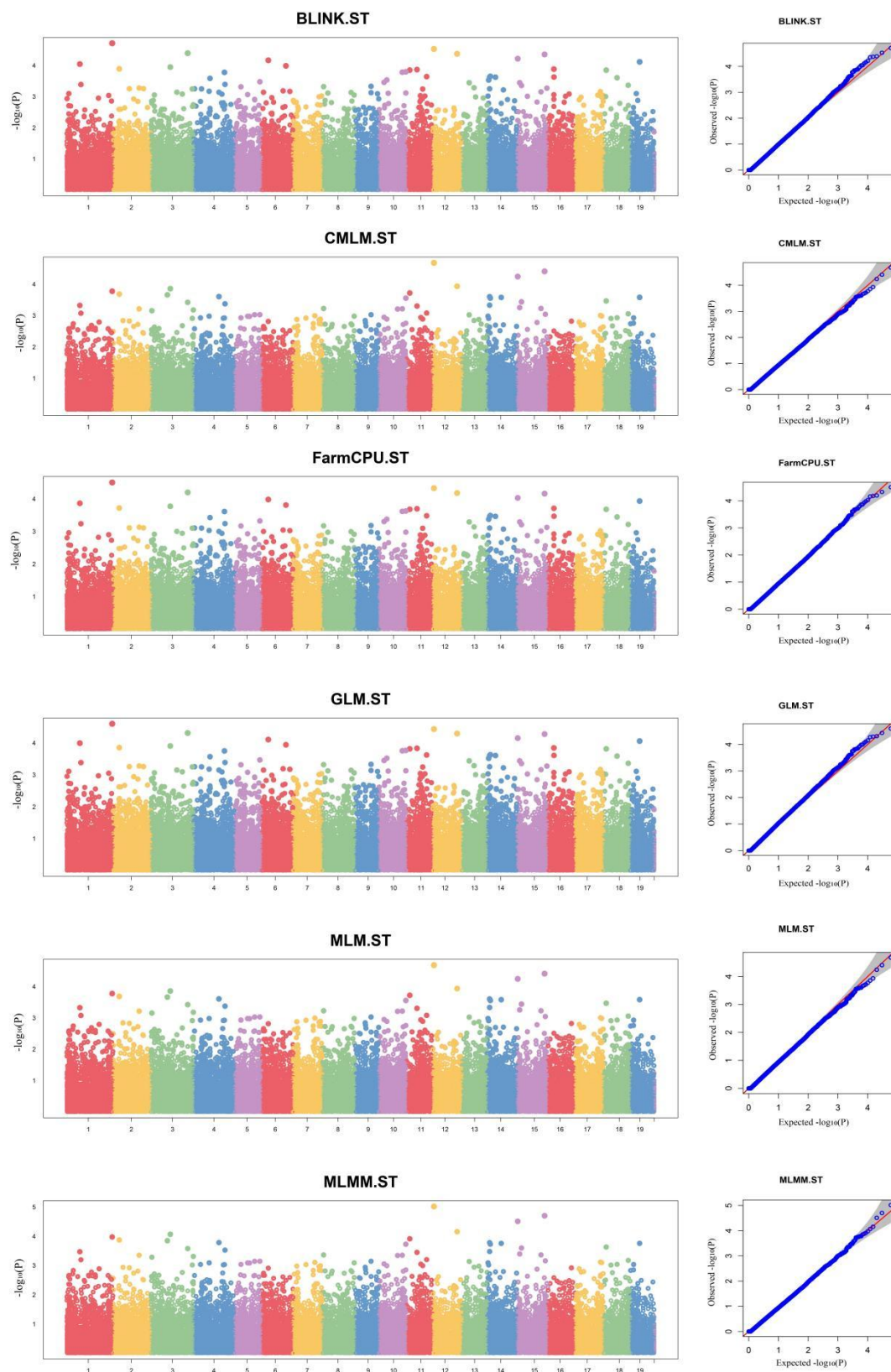

Fig. S17 Figure of the GWAS results for ST using InDels.

Supplement: Supplementary file 1 — Supplementary data to this article can be found online. [file forres-0025-0001-S1.zip › 10.48130_forres-0025-0001-Suppl-FigureS17.pdf]

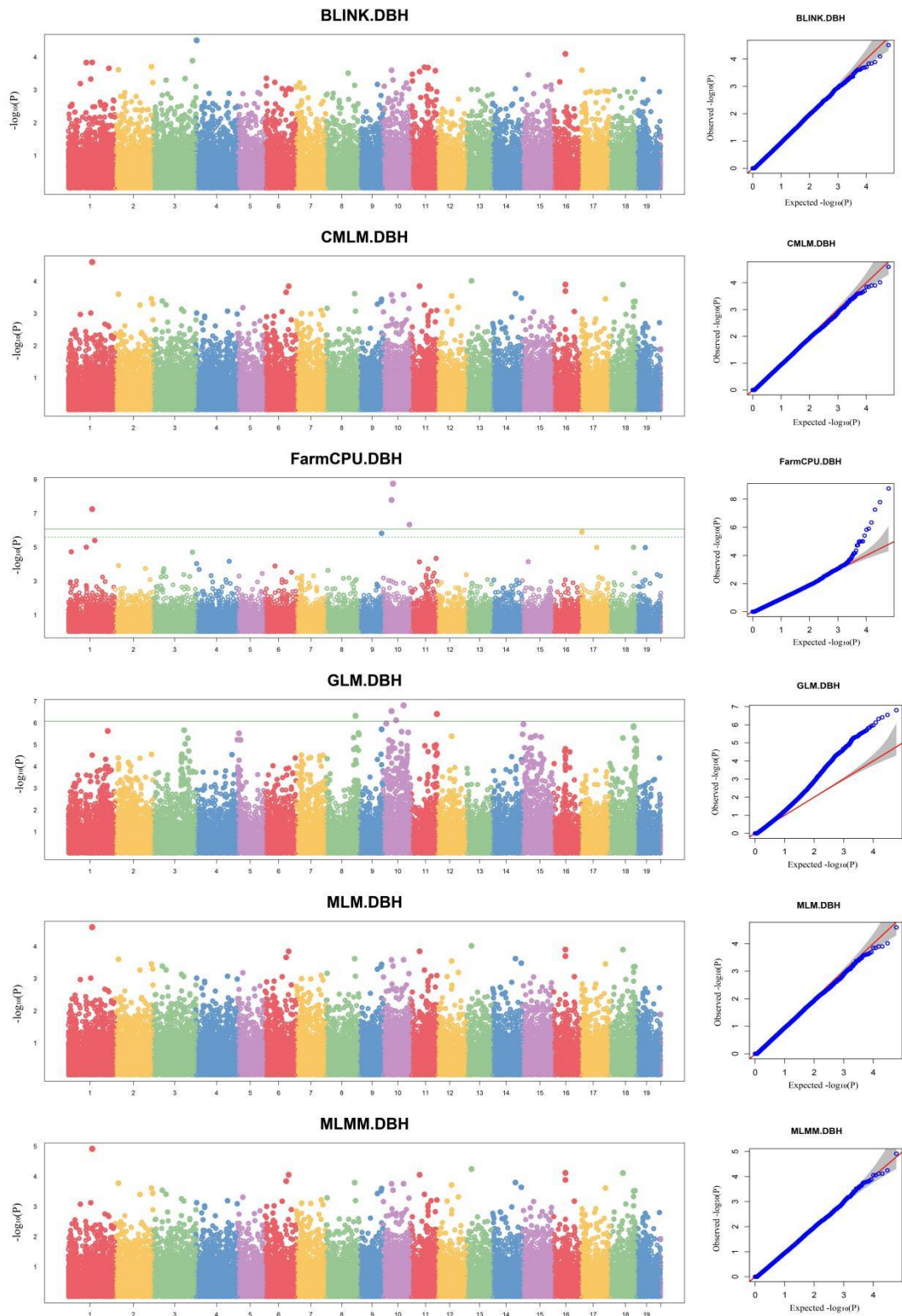

Fig. S18 Figure of the GWAS results for DBH using InDels.

Supplement: Supplementary file 1 — Supplementary data to this article can be found online. [file forres-0025-0001-S1.zip › 10.48130_forres-0025-0001-Suppl-FigureS18.pdf]

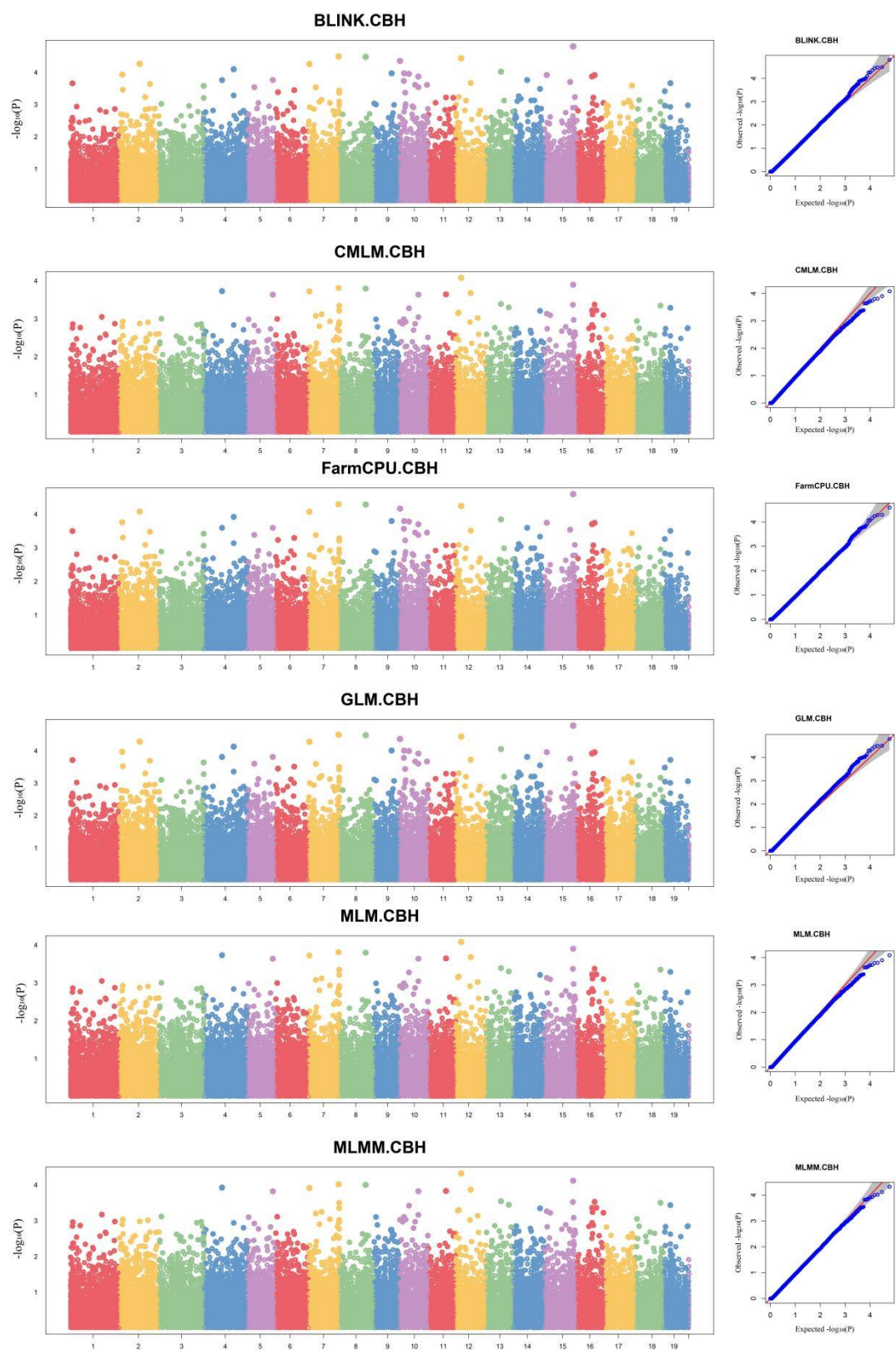

Fig. S19 Figure of the GWAS results for CBH using InDels.

Supplement: Supplementary file 1 — Supplementary data to this article can be found online. [file forres-0025-0001-S1.zip › 10.48130_forres-0025-0001-Suppl-FigureS19.pdf]

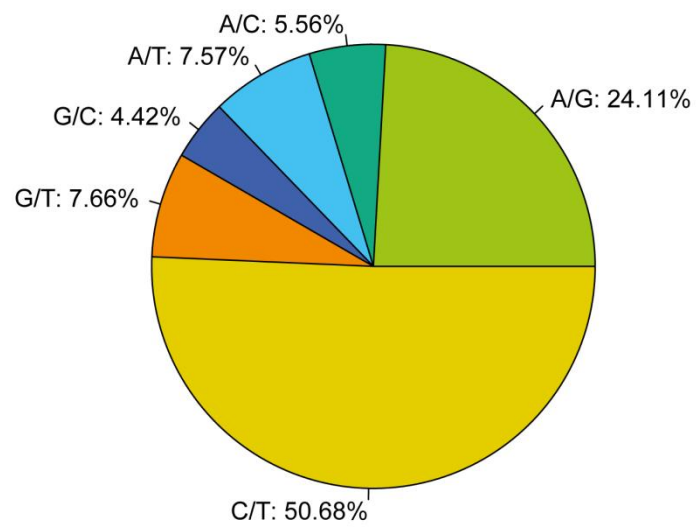

Fig. S2 Six types of nucleotide substitution pie charts.

Supplement: Supplementary file 1 — Supplementary data to this article can be found online. [file forres-0025-0001-S1.zip › 10.48130_forres-0025-0001-Suppl-FigureS2.pdf]

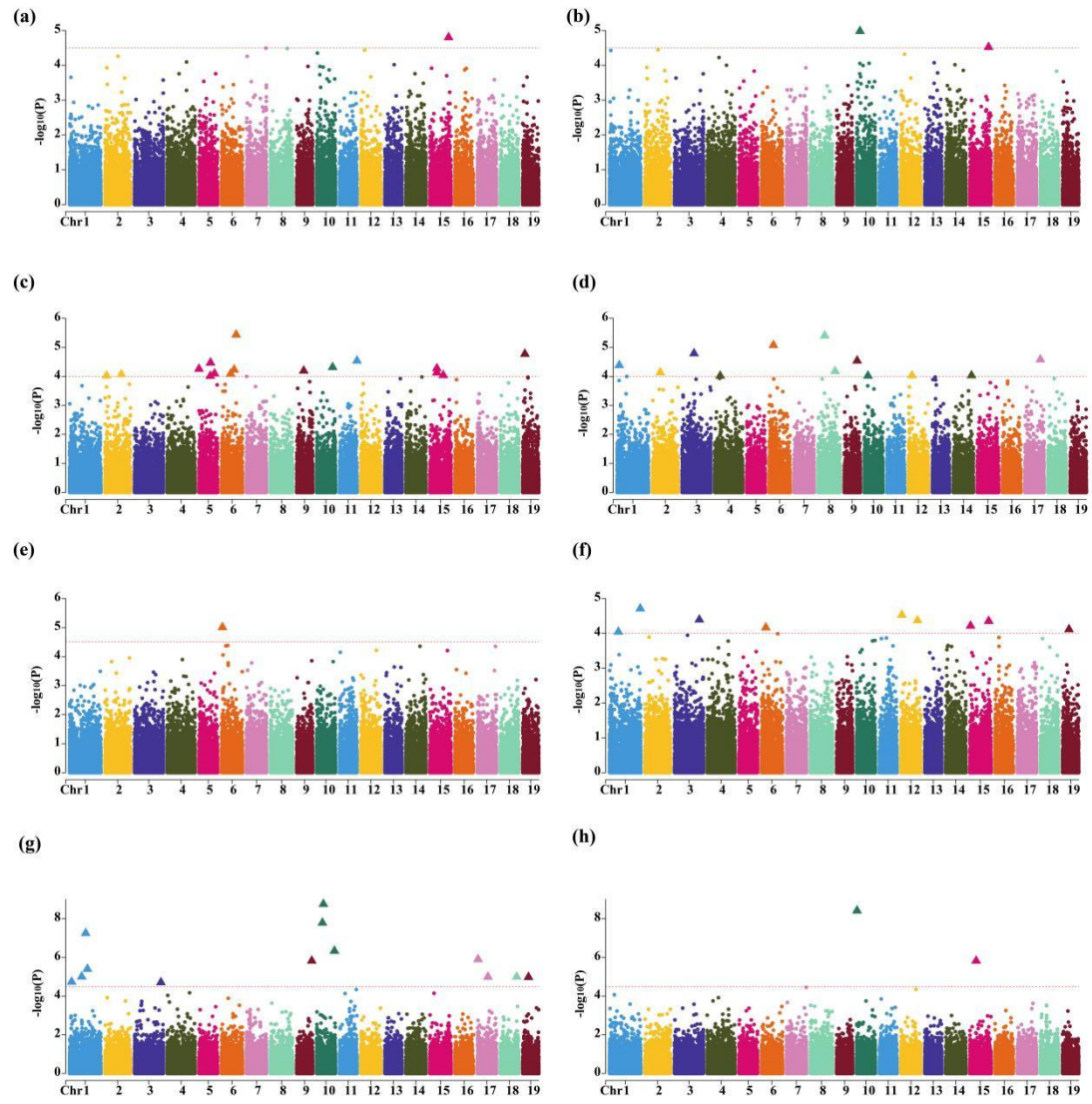

Fig. S3 Manhattan maps for GWAS analysis using InDel (a) CBH; (b) CLR; (c) BA; (d) FK; (e) NB; (f) ST; (g) DBH; (h) H.

Supplement: Supplementary file 1 — Supplementary data to this article can be found online. [file forres-0025-0001-S1.zip › 10.48130_forres-0025-0001-Suppl-FigureS3.pdf]

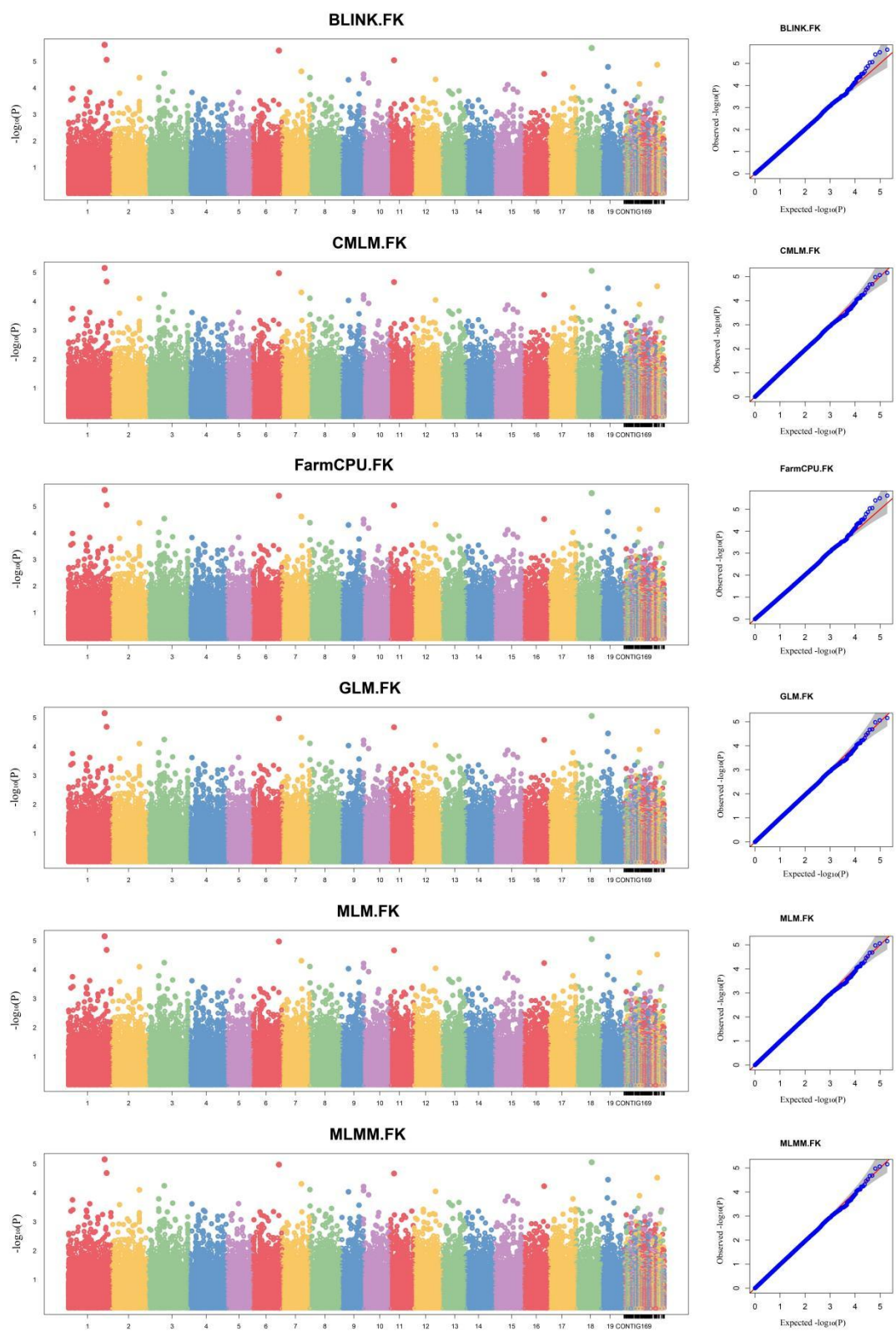

Fig. S4 Figure of the GWAS results for FK using SNPs.

Supplement: Supplementary file 1 — Supplementary data to this article can be found online. [file forres-0025-0001-S1.zip › 10.48130_forres-0025-0001-Suppl-FigureS4.pdf]

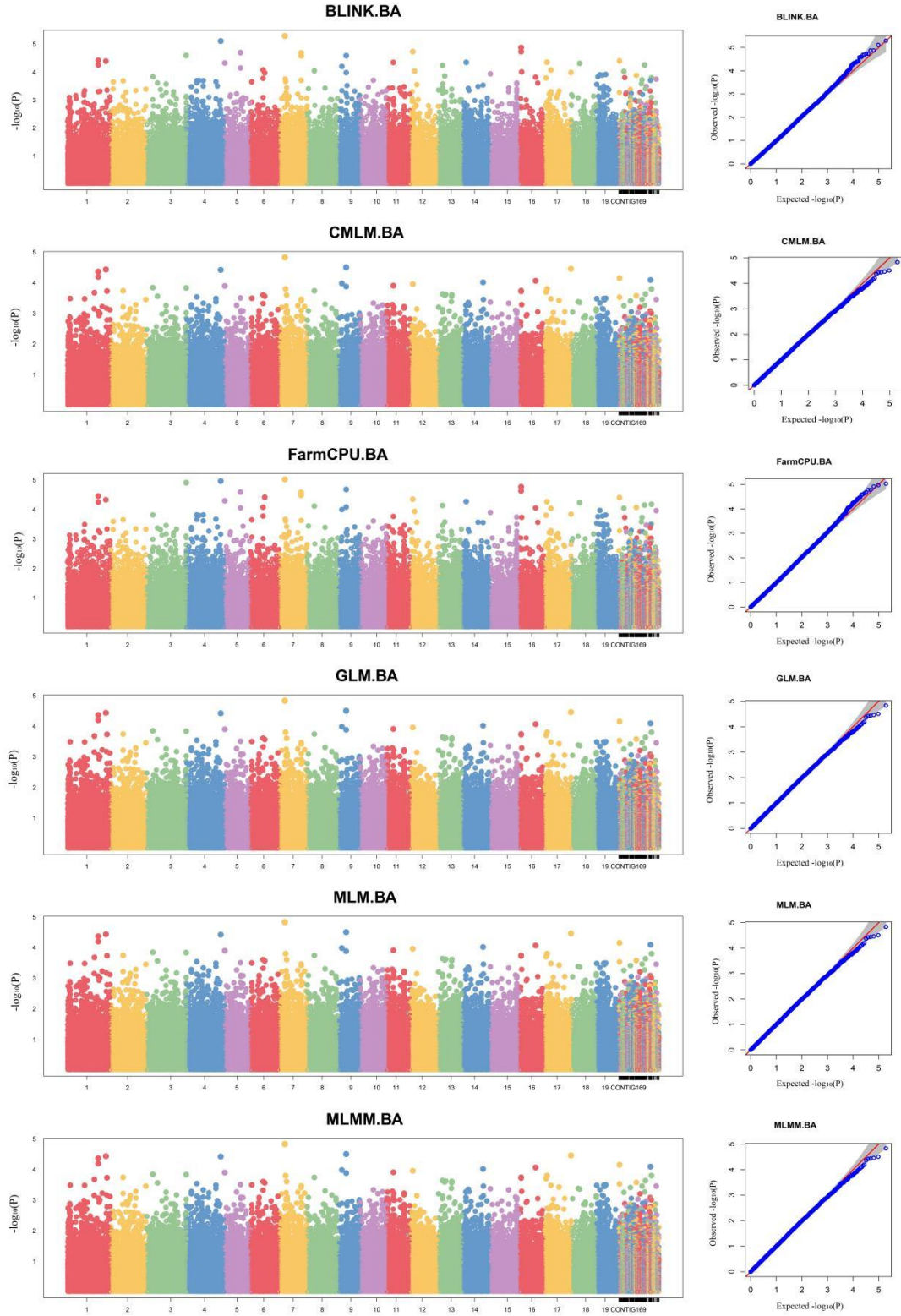

Fig. S5 Figure of the GWAS results for BA using SNPs.

Supplement: Supplementary file 1 — Supplementary data to this article can be found online. [file forres-0025-0001-S1.zip › 10.48130_forres-0025-0001-Suppl-FigureS5.pdf]

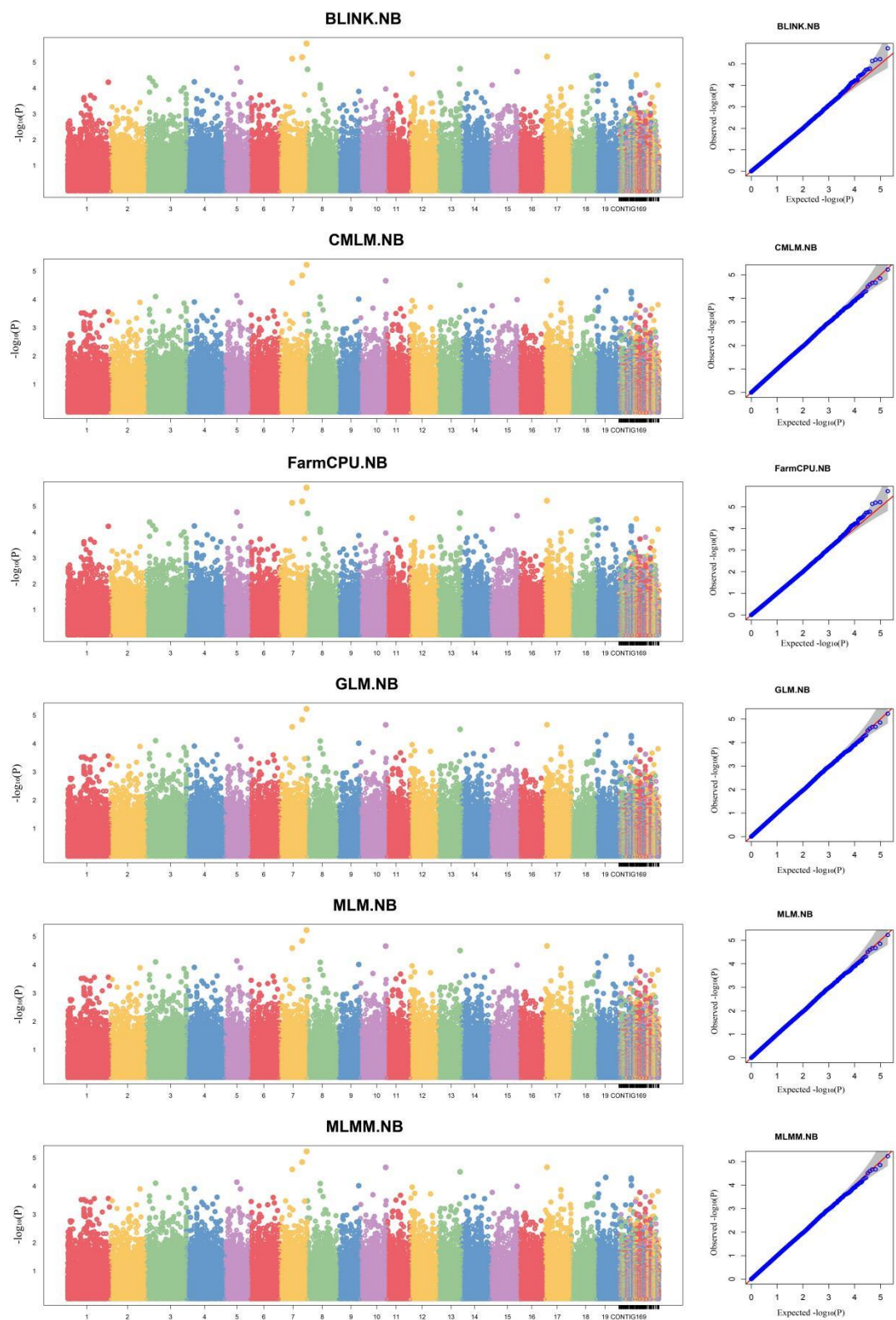

Fig. S6 Figure of the GWAS results for NB using SNPs.

Supplement: Supplementary file 1 — Supplementary data to this article can be found online. [file forres-0025-0001-S1.zip › 10.48130_forres-0025-0001-Suppl-FigureS6.pdf]

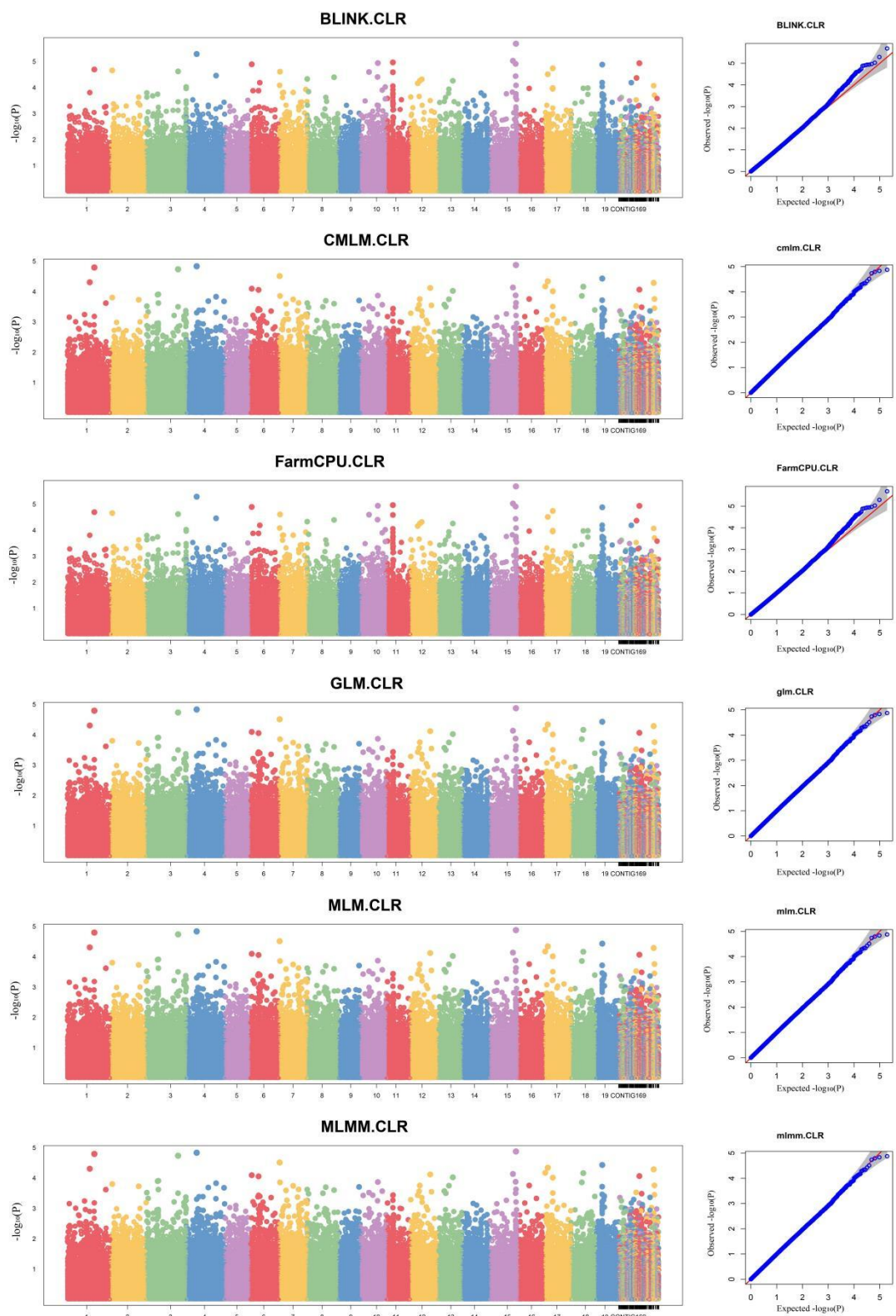

Fig. S7 Figure of the GWAS results for CLR using SNPs.

Supplement: Supplementary file 1 — Supplementary data to this article can be found online. [file forres-0025-0001-S1.zip › 10.48130_forres-0025-0001-Suppl-FigureS7.pdf]

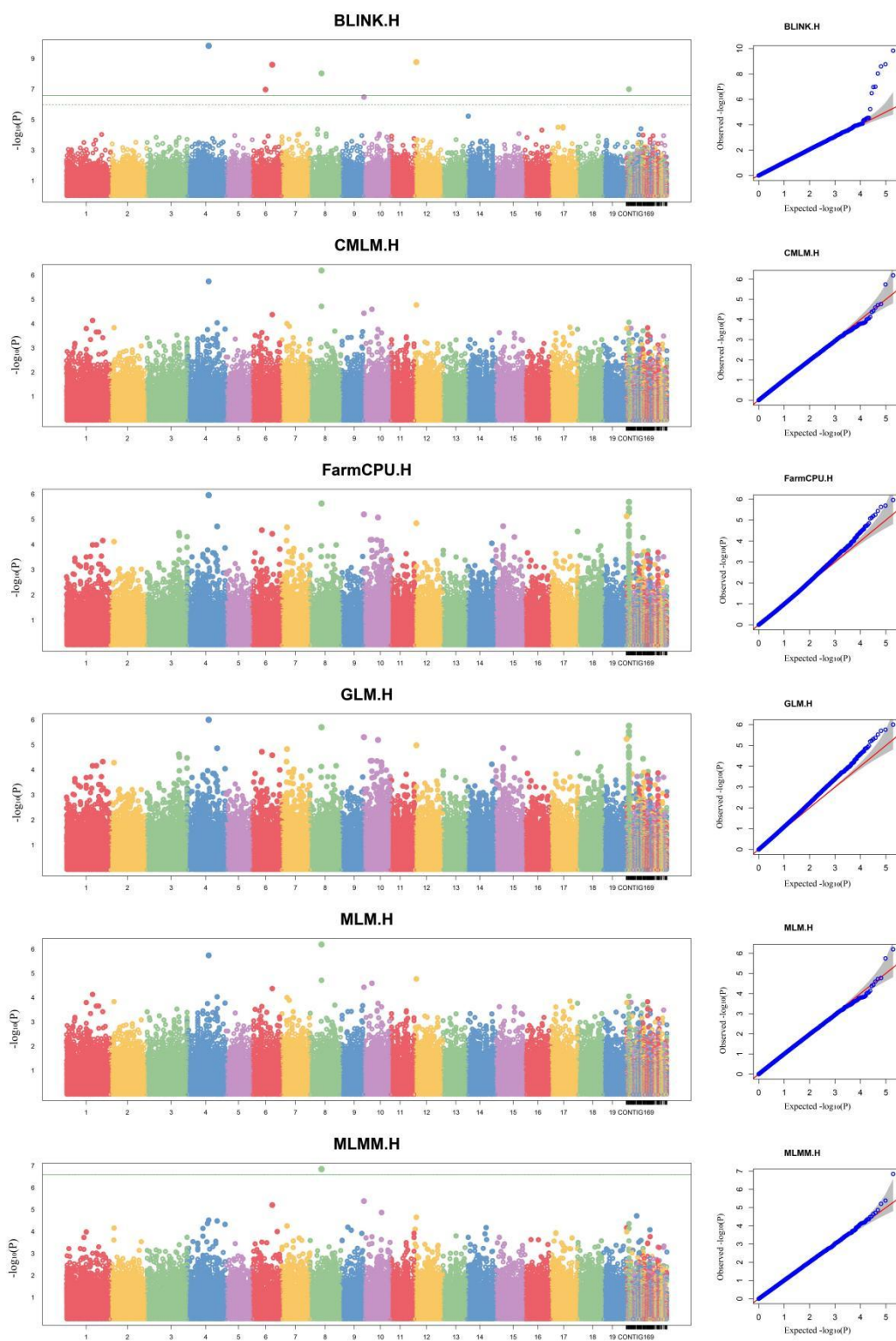

Fig. S8 Figure of the GWAS results for H using SNPs.

Supplement: Supplementary file 1 — Supplementary data to this article can be found online. [file forres-0025-0001-S1.zip › 10.48130_forres-0025-0001-Suppl-FigureS8.pdf]

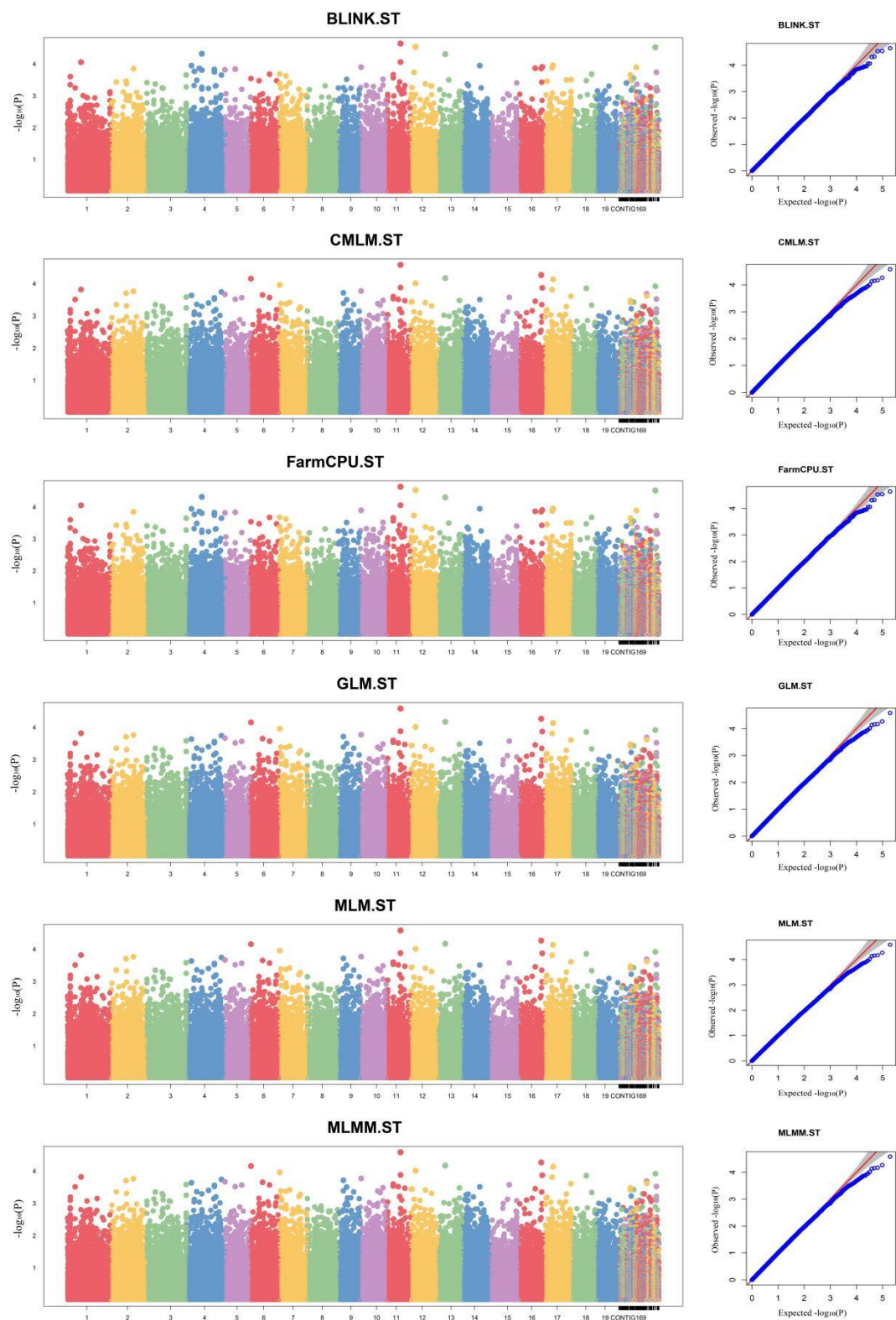

Fig. S9 Figure of the GWAS results for ST using SNPs.

Supplement: Supplementary file 1 — Supplementary data to this article can be found online. [file forres-0025-0001-S1.zip › 10.48130_forres-0025-0001-Suppl-FigureS9.pdf]
